# Operational building datasets from multipurpose buildings in Denmark and Switzerland: High-resolution energy use and room measurements

**DOI:** 10.1016/j.dib.2026.112719

**Published:** 2026-03-24

**Authors:** Simon Pommerencke Melgaard, Thomas Juul, Rasmus Lund Jensen, Alessandro Tell, Gabriele Humbert, Reto Fricker, Sascha Stoller, Marco Kreyenbuehl, Philipp Heer, Binod Koirala

**Affiliations:** a Department of the Built Environment, Aalborg University, Thomas Manns vej 23, 9220 Aalborg East, Denmark; b Urban Energy Systems Lab, Swiss Federal Laboratories for Materials Science and Technology, Überlandstrasse 129, 8600 Dübendorf, Switzerland

**Keywords:** HVAC, IEQ, Heating, Electricity, Occupancy, Energy meters

## Abstract

The dataset comprises Building Management System (BMS) data from an educational building located on the main campus of Aalborg University in Denmark, as well as from Empa’s NEST (Next Evolution in Sustainable Building Technologies) demonstrator building in Switzerland. The buildings contain main and sub-meters for all equipment using electricity or thermal energy. The equipment using thermal energy includes air handling units (water-based heating coils), space heating (floor heating, radiators, and ceiling heating), and domestic hot water (heat exchangers). Besides the energy data, room data, such as temperature, CO2 concentration, occupant presence, radiator valve opening, and ventilation damper opening, are included for all rooms in the buildings.

The data spans 6 to 28 months, depending on the building and the measurement points. The data was collected as raw data with a time resolution between 1 and 10 min. The dataset is expected to be useful for various applications, including model calibration, machine learning, and occupant analysis.

Specifications Table

**Table:** 

Subject	Engineering & Materials science
Specific subject area	Building energy use, indoor climate measurements, and HVAC system operational data
Type of data	Tables (.csv format in zip files) with raw data Tables (.xlsx format) with overview of measurement points Images (.jpg and .pdf format in zip files) with supporting data (data availability, plan drawings, and system schematics)
Data collection	For the AAU pilot building, the data was collected using the Building Management System software EcoStruxure from Schneider Electric, feeding into a local PostgreSQL database with TimescaleDB extension for long-term storage. For the NEST Building, data was collected using OPC-UA and postgres SQL database for collecting data.
Data source location	The AAU pilot building houses the Department of the Built Environment, and is located at Thomas Manns vej 23, 9220 Aalborg Øst, Denmark, at the following coordinates: 57 °00′52.2″N 9 °58′23.5″E. Empa’s NEST demonstrator building is located at Überlandstrasse 129, 8600 Dübendorf, Switzerland, at the following co-ordinates: 47 °24′10.25″N 8 °36′44.5″E.
Data accessibility	Repository name: Zenodo Data identification number: 10.5281/zenodo.19019863 Direct URL to data: https://zenodo.org/records/19019863
Related research article	none

## 1 Value of the Data

• The dataset will be one of only a few high-resolution openly available building datasets, with the datapoints having a time resolution of between 1 and 10 min, and most having roughly 1.5 years of AAU data and 9 months of NEST data. Thus allowing researchers without access to high-quality building data to test or validate their ideas.
• The dataset covers all electricity and heating energy main and submeters along with operational parameters for air handling units, radiator, ceiling heating, and floor heating systems, and domestic hot water systems. Besides the different parts of the building's Heating, Ventilation, and Air-Conditioning (HVAC) system, the main room parameters are also provided for all rooms in the building. In total, 709 individual datapoints are provided for AAU building and 334 data points are provided for the NEST buildings. Thus, allowing the development of monitoring methods for use with highly detailed data, as well as systems with less data, which would be the case for most buildings.
• Alongside the dataset, the different installations and schematics are described for both the AAU and NEST buildings, with two Air Handling Units (AHUs) at AAU being highly detailed. This comprehensive documentation enhances transparency and reproducibility, enabling researchers and practitioners to better interpret the dataset, validate modelling assumptions, and apply the insights to comparable building energy systems.
• Includes different building types: university/research facilities, residential, and office. This could be used for comparative analysis of the differences and similarities between various building types.
• The dataset should prove useful to researchers working within energy system modelling, occupancy detection, energy analysis, forecasting of building performance, and Indoor Environmental Quality (IEQ) analysis due to the high resolution and coverage of the dataset.

## 2 Background

The datasets were collected as part of the HORIZON Europe project “HEATWISE” [1] where the focus was on the building's and systems' heating needs. To analyze and interpret the data, the electricity, heating, room-level, system operations, and outdoor data were also collected. As the dataset was already collected in the project, our goal is always to make as much data as possible publicly available to provide opportunities to other researchers who might lack access to high-resolution datasets.

Due to the scope of the work carried out in “HEATWISE”, the dataset does not cover all data points available in the AAU and NEST pilot; it is, therefore, possible to contact either simonpm@build.aau.dk or rlje@build.aau.dk to get access to the updated or live data for all the data points for the AAU pilot presented in this paper. Similarly, for the NEST pilot, it is possible to contact either reto-fricker@empa.ch or binod.koirala@empa.ch. NEST data are available after registering and requesting access to the NEST database (see https://wiki.nestcloud.ch).

## 3 Data Description

The dataset described in this article can be found here [2].

The dataset includes two pilot cases, with the abbreviations related to the pilots being available in Table 1. The two pilots are the Aalborg University (AAU) educational building and the Swiss federal laboratories for material science and technology (Empa) multi-purpose building named Next Evolution in Sustainable Building Technologies (NEST). The NEST building contains several individual building units, with those included in this dataset being named: Urban Mining and Recycling (UMAR), Sprint, Digital Fabrication (DFAB), and High Performance and Low Emissions (HiLo).

**Table 1:** Abbreviations used in the paper.

Abbreviations (project and pilot)	Description
HEATWISE	Holistic Energy Management and Thermal Waste Integrated System for Energy Optimisation
AAU	Aalborg University
Empa	Swiss federal laboratories for material science and technology
NEST	Next Evolution in Sustainable Building Technologies
UMAR Sprint DFAB HiLo	Urban Mining and Recycling (residential unit at NEST) A flexible and rapidly reconfigurable office unit at NEST Digital Fabrication (residential unit at NEST) High Performance and Low Emissions (office unit at NEST)
**Abbreviations (general)**	
DHW	Domestic hot water
CO2	Carbon dioxide
AKS	Equipment identification system
HVAC	Heating, ventilation, and air-conditioning
PV AHU	Photovoltaics Air handling unit
DH	District Heating
HC	Heating coil
HX	Heat exchanger
BMS	Building management system
VAV	Variable air volume
CAV	Constant air volume
PIR	Passive infrared
MTE	Medium temperature
HTE	High temperature
TABS	Thermally activated building systems
EPC	Energy performance certificate

This article is structured so anything related to the data, such as location of the different zones, energy metering schematic, and similar is found in this section, while information regarding the more technical aspects of specific systems, components, and similar can be found in the section Experimental Design, Materials and Methods.

The dataset is split into three main parts and two supporting parts as follows:

- Main parts
  ○ Data_overview.xlsx
    ▪ This file describes the data points available in the two pilot buildings included, along with sensor accuracy.
    ▪ It also includes the plan drawings and schematics of the AAU pilot
  ○ AAU_pilot_data_X.zip
    ▪ These zip folders includes the individual data files (.csv) of the AAU pilot
    ▪ The X denotes the category of data, such as ventilation, electricity, etc.
    ▪ The name of each file corresponds to those found in the “Data_overview.xlsx” files, in the tab “AAU_pilot” and column “Data in file”.
  ○ NEST_pilot_data.zip
    ▪ This zip folder includes the individual data files (.csv) of the NEST pilot
    ▪ The name of each file indicates the building unit it belongs to and which type of system/meter it contains data points for
- Supporting parts
  ○ AAU_pilot_available_data_images.zip
    ▪ This zip folder contains images showing the availability of each data point
    ▪ The images can only be seen in the folder, not in this article
    ▪ The name of each image corresponds to the name from the data files in “AAU_pilot_data.zip”
  ○ NEST_pilot_images.zip
    ▪ This zip folder contains images showing the availability of each data point, along with the plan drawings and schematics of the NEST pilot
      • The plan drawings and schematics are described in this article
      • The data availability images can only be seen in the folder, not in this article
    ▪ The name of the data availability images corresponds to the name from the data files in “NEST_pilot_data.zip”

### 2.1 Overview file “data_overview”

The overview file contains the full overview of each datapoint, what it covers, and in which of the data files it can be found.

In the overview file, there are 9 tabs:

- Explanation of inputs
  ○ Contains a brief explanation of the columns in the tab “AAU_pilot”
- AAU_pilot
  ○ Contains all the information on where to find the different measurement points, what they mean, what unit they are measured in, and what the sensor accuracy is
- AAU_plans
  ○ Contains the distribution grid schematics for the electricity and heating networks in the building as well as which systems cover which zones in the building
- AAU_Space_heating
  ○ Shows an example of how the space heating systems are built up, and where the different measurements are taken from in it
- AAU_Domestic_hot_water
  ○ Shows an example of how the domestic hot water systems are built up, and where the different measurements are taken from in it
- AAU_Vent_KOMF01
  ○ Shows how the KOMF01 air handling unit is built up, and where the different measurements are taken from in it, along with specifications on the major components
- AAU_Vent_KOMF02
  ○ Shows how the KOMF02 air handling unit is built up, and where the different measurements are taken from in it, along with specifications on the major components
- AAU_Misc_Door01
  ○ Shows how the Door01 heat blanket system is built up, and where the different measurements are taken from in it
- NEST_pilot
  ○ Contains all the information on where to find the different measurement points, what they mean, what units they are measured in, and what the sensor accuracy is

In the tabs “AAU_pilot” and “NEST_pilot”, the units are defined according to [3], as well as with their corresponding SI unit. The reason for this was to avoid ambiguity across different disciplines that may use the same notation for different units. Not all of the units used could be found directly in the standard; some are therefore mentioned with either a multiplication (*) or division (/) symbol. If this is the case, the conversion factor provided in the column “Unit conversion factor” needs to be multiplied by the data to obtain the provided unit given in the column “Unit”. As an example the water flow data is recorded in litre/second, this is not an available unit, therefore litre/minute has been provided along with the conversion factor 60, as litre/second multiplied with 60 s/minute gives litre/minute. Once the conversion factor has been applied, a second conversion factor from the column “Unit (SI)” can be used to transform the data point to the corresponding SI unit.

Besides the units, the two tabs also contain the sensor types for each measurement point, along with a measurement range, and accuracy.

### 2.2 AAU pilot

The dataset is comprised of 33 individual data files (.csv format), an overview file called “data_overview” (.xlsx format), and a zip folder called “Available_data_images.zip” containing the images with the data availability of the different data points. The images are separated into their own subfolders according to the type of data they describe. An overview of the 33 data files can be seen in Table 2.

**Table 2:** Overview of AAU pilot data files along with their category. For the file “electricity”, it is located in “Main meters”, but it contains all the individual electricity meters alongside the main meter.

Main meters	Space heating	Domestic hot water	Ventilation	Miscellaneous / Outdoor	Rooms
FO01	GV01	VVB01_basement	KOMF01	Door01	Indoor_floor_1
electricity	GV02	VVB01_0_east	KOMF02	outdoor	Indoor_floor_2_part_1
	VA01	VVB02_0_west	KOMF03		Indoor_floor_2_part_2
	VA02	VVB01_1_east	LAB1		Indoor_floor_3_part_1
		VVB02_1_west	LAB2		Indoor_floor_3_part_2
		VVB01_2_east	P-KLIMA		Indoor_floor_4
		VVB02_2_west	P-FUND		
		VVB01_3_east	P-VAND		
		VVB02_3_west	P-VAERK		
			VU23		

For all the data, the timestamp is provided in the following format “YYYY-MM-DD hh:mm:ss+hh:mm” with the timezone corresponding to Danish local time, which is either UTC+1 (CET) or UTC+2 (CEST). The data is generally available in the period from 2023 to 07–01 to 2024–07–01, but many measurement points also have data going all the way back to 2022–03–01.

#### 3.2.1 Main meters

**Fig. 1:**
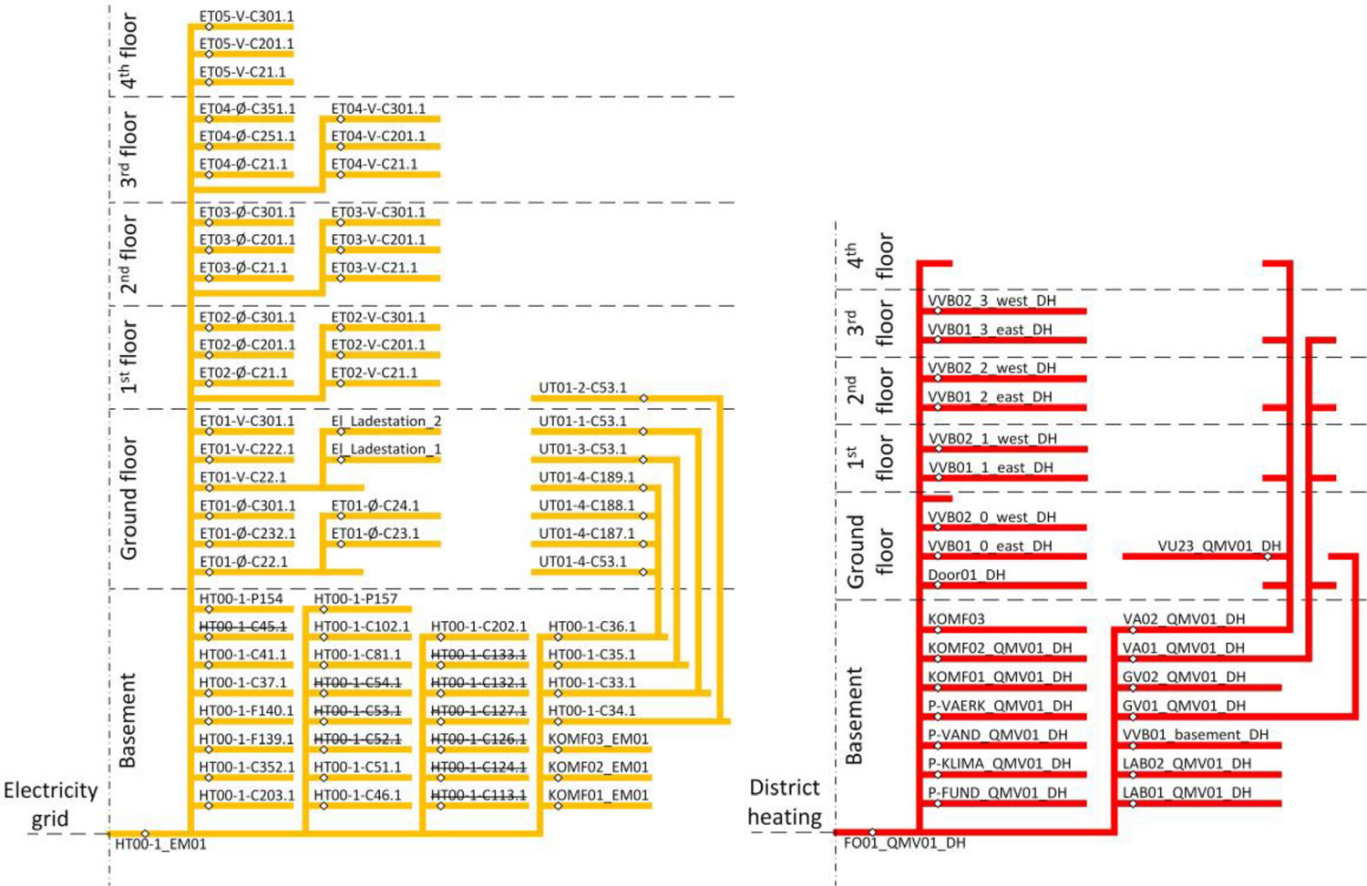
The left figure shows the electricity network inside the building with both the main meter and sub meters. The right figure shows the heating network inside the building with both the main meter and sub meters. On both figures, the points on the lines mark the locations of the meters along with their group ID. These figures only indicate the floor, not the location in the building.

The dataset includes all the available main and submeters for both electricity and heating energy. For the electricity meters, the variable measured is the cumulative energy use. An overview of the location of each meter can be seen in Fig. 1, where each end of the yellow lines indicates an electrical draw point. This is especially important to be aware of when dealing with the submeters, as some submeters may have further submeters. For example, HT00–1-C34.1 has both an electrical draw point and a submeter (UT01–2-C53.1). It should also be noted that some electrical meters do not exist anymore (when crossed out), but for completeness of the overview, they are included. An overview of the different periods of available data for the electricity meters can be seen in the file” electricity_2022_1__2024_7.png” in the “AAU_pilot_available_data_images” folder’s “electricity” subfolder. In general, the electricity submeters are lacking significant periods of data, with most though available in the period from 2024 to 01–01 to 2024–07–01, and the main electricity meter being available from 2022 to 10–01 to 2024–07–01.

For the heating energy meters, they can be seen in Fig. 1, with all the submeters being further described in the following sections. The main meter is located on the district heating (DH) connection to the building, which is made as a direct connection, meaning there is no heat exchanger separating the building from the DH grid. The variables of the heating meters include supply temperature, return temperature, flow (both instantaneous and cumulative), as well as energy use and power. For the main heating meter, its available data can be seen in the file” FO01_2022_1__2024_7.png” in the “AAU_pilot_available_data_images” folder’s “Main heat meter” subfolder. The main heating meter has data in the period from 2022 to 10–01 to 2024–07–01.

#### 3.2.2 Space heating

**Fig. 2:**
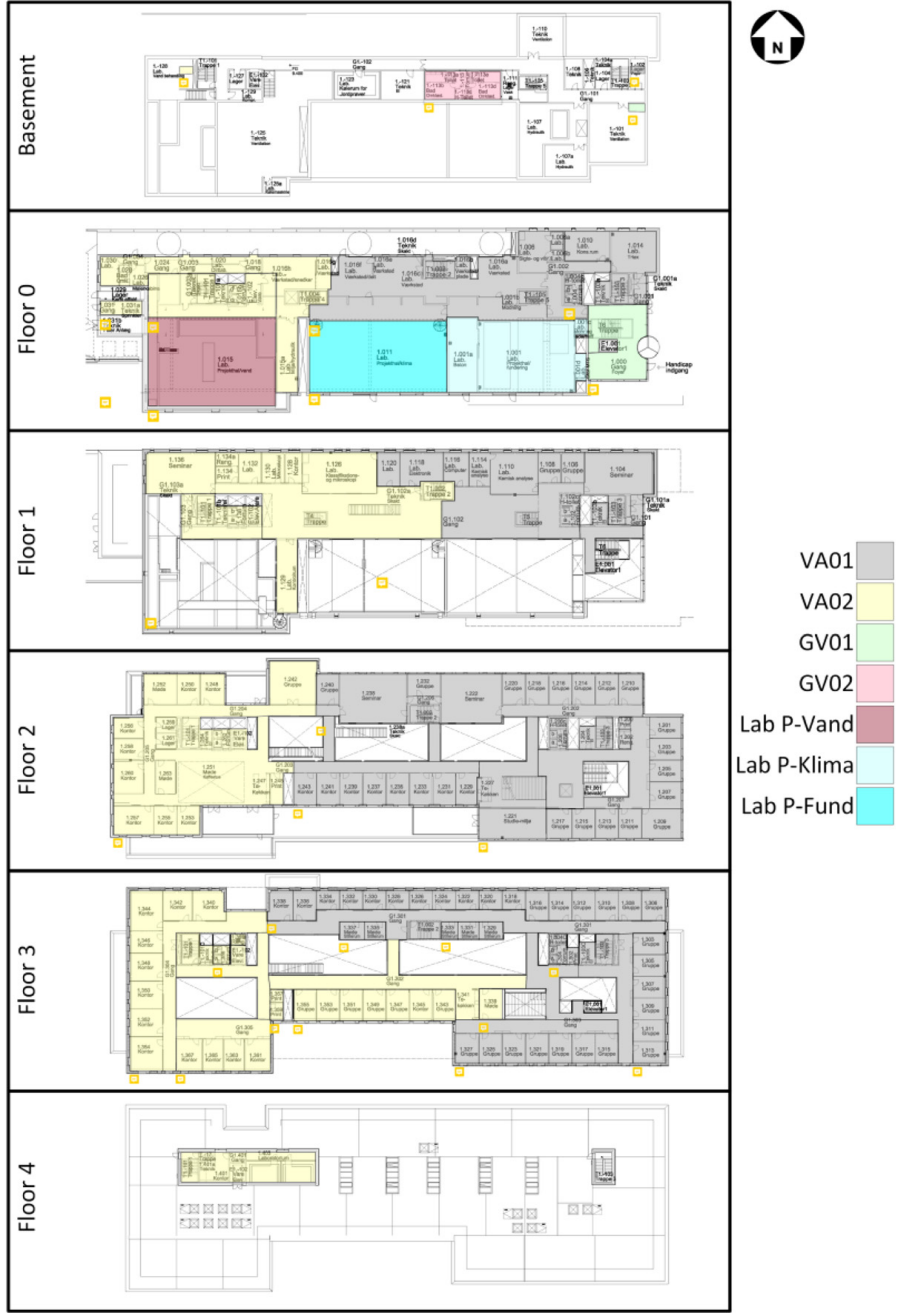
Space heating zones covered by the different systems. The three Lab zones do not have submetering; they are therefore not available in the dataset. The original image is from the Waste heat potential report by Melgaard et al. (2024) [4] and is reused with permission of the authors.

The space heating is comprised of seven different circuits, but only four of them (VA01/02 and GV01/02) have submetering. An overview of the rooms they cover can be seen in Fig. 2.

The space heating submeters have the variables as described in the main meters section, along with the supply water temperature of the space heating as can be seen in Fig. 27. An overview of the available data for space heating can be seen in the files in the “AAU_pilot_available_data_images” folder’s “Space heating” subfolder. In general, the space heating measurements are all at least available in the period from 2023 to 01–01 to 2024–07–01.

#### 3.2.3 Domestic hot water

**Fig. 3:**
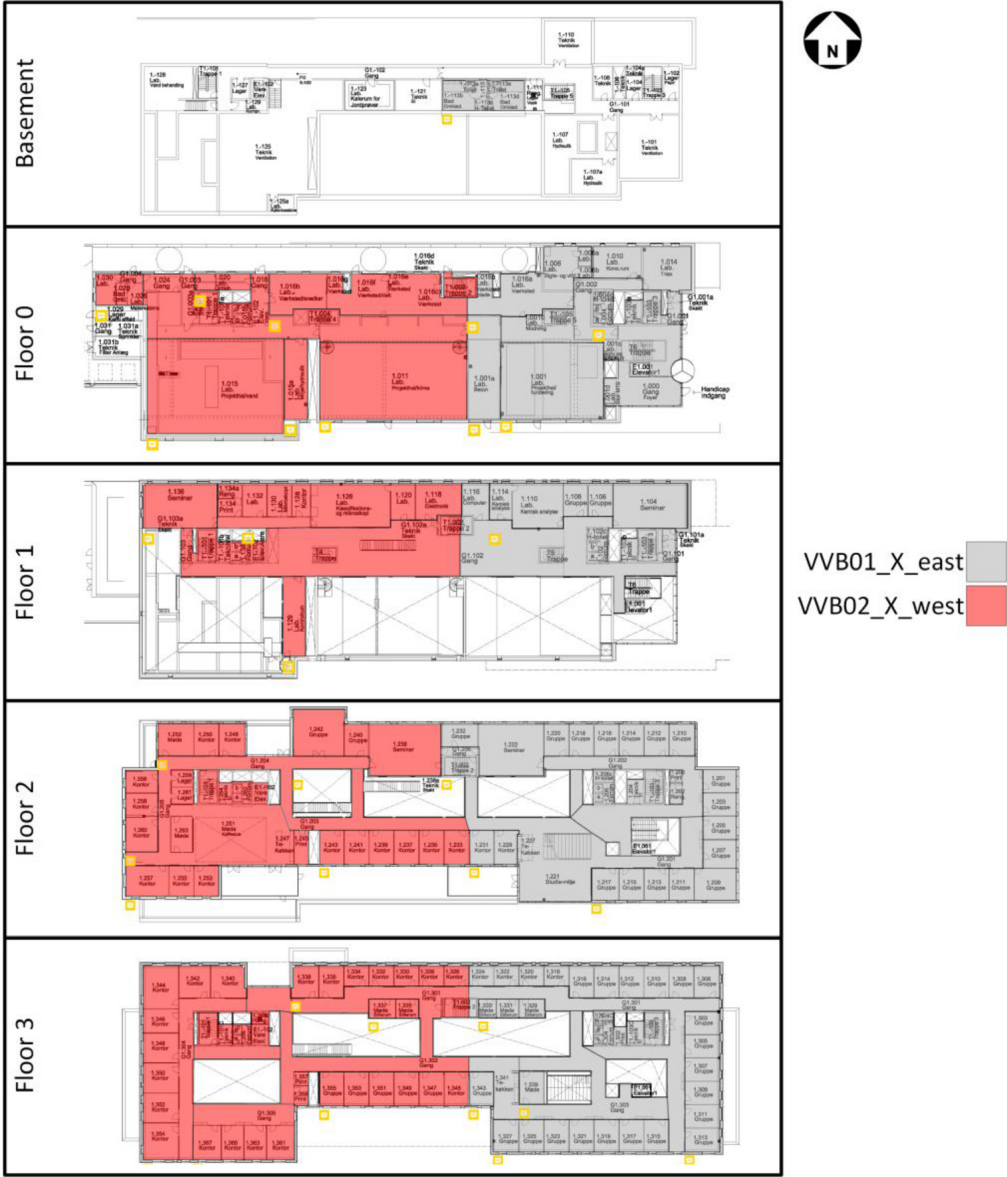
Domestic hot water zones. Each floor has two DHW heat exchangers except for the basement, which has a single heat exchanger. The “X” in the naming refers to the floor number. The original image is from the Waste heat potential report by Melgaard et al. (2024) [4] and is reused with permission of the authors.

The domestic hot water (DHW) system in the building is based on each floor having two heat exchangers (except for the basement, which only has one). For each of the DHW circuits, the DH measurements are available along with the supply temperature, circulation temperature, and cold water flow to the system, as can be seen in Fig. 28. An overview of the different DHW zones can be seen in Fig. 3, while the available data for each zone can be seen in the files in the “AAU_pilot_available_data_images” folder’s “DHW” subfolder. In general, the DHW measurements are somewhat lacking, with almost all measurement points being available in the period from 2024 to 01–01 to 2024–07–01, though many are available going further back.

#### 3.2.4 Ventilation

**Fig. 4:**
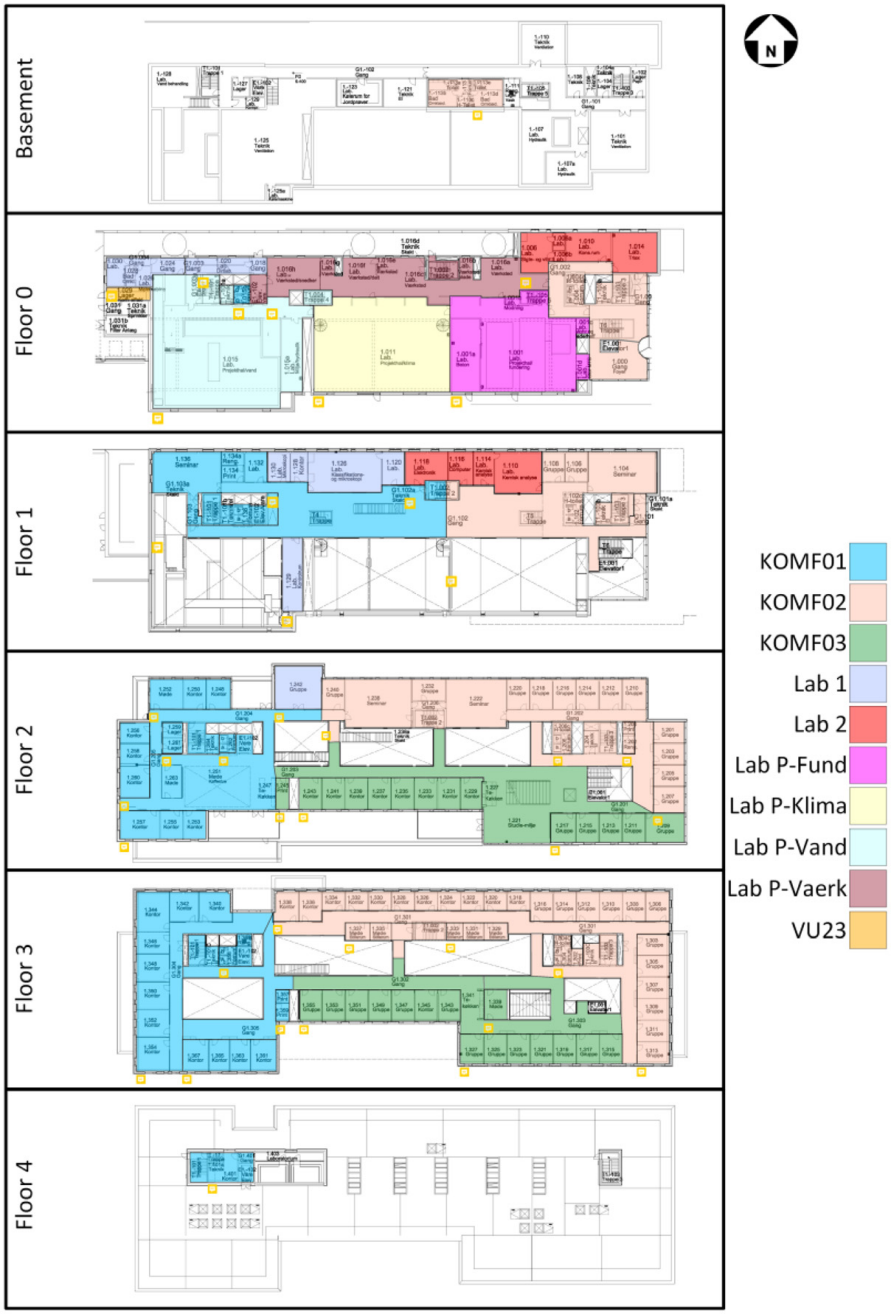
Zones covered by the different air handling units. The original image is from the Waste heat potential report by Melgaard et al. (2024) [4] and is reused with permission of the authors.

The air handling units (AHUs) in the building can be primarily categorized into two types: comfort ventilation and laboratory ventilation. The comfort ventilation units consist of the “KOMF01”, “KOMF02”, and “KOMF03” units. The first two of these units have significantly more measurement points recorded than the last unit, with a schematic and notation of the different measurement points being available in Fig. 29, Fig. 30. The specifications for the two units can further be found in Table 4. For “KOMF03” and all the laboratory units, there is only data available for the heating energy meter on the heating coil. The different zones covered by the individual AHU can be seen in Fig. 4, while the available data periods for each data point can be seen in the files in the “AAU_pilot_available_data_images” folder’s “Ventilation” subfolder. In general, the ventilation measurements are available for the period 2023–01–01 to 2024–07–01, except for the “P-FUN”, “P-VAERK”, “P-VAND”, and “VU23” which are only available in the period 2024–01–01 to 2024–07–01.

#### 3.2.5 Miscellaneous

The miscellaneous category contains one type of system, which is the air curtain for the main revolving door located on the eastern side of floor 0. The schematic and measurement points of the air curtain can be seen in Fig. 31, while the available data can be seen in the file “Door01_2022_1__2024_7.png” in the “AAU_pilot_available_data_images” folder’s “Miscellaneous” subfolder. The miscellaneous measurements are available for the period 2024–01–01 to 2024–07–01, with some data further back.

#### 3.2.6 Outdoor

The outdoor measurements include the wind speed, the outdoor temperature, and three solar radiations for the east, south, and west directions. The period of available data can be seen in the file “outdoor_2022_1__2024_7.png” in the “AAU_pilot_available_data_images” folder’s “Outdoor” subfolder. The outdoor measurements are available for the period 2022–06–01 to 2024–07–01.

#### 3.2.7 Rooms

**Fig. 5:**
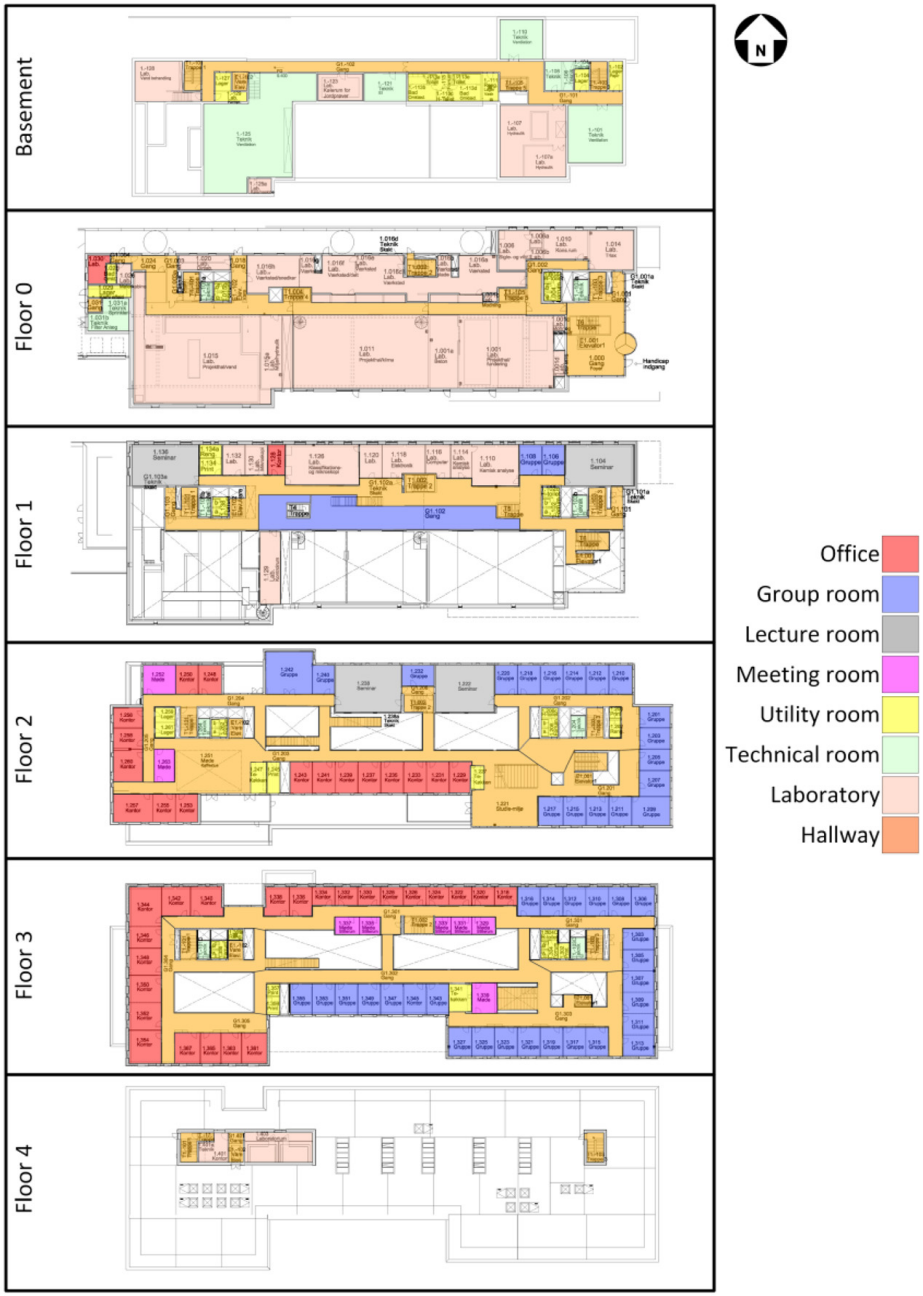
Room types in the building. The original image is from the Waste heat potential report by Melgaard et al. (2024) [4] and is reused with permission of the authors.

For the room measurements, five different measurement points are generally available for most rooms, these are; room temperature, carbon dioxide (CO2) concentration, opening percentage of the radiator motor valve, opening percentage of the ventilation dampers in the room, and presence of people in the room. The different types of rooms included in the measurement data are the offices for employees, group rooms for students, meeting rooms, and lecture rooms. For this reason, no rooms from the basement and floor 0 are included, but they are shown for the sake of completeness in Fig. 5.

Most of the room measurements are available for at least a six month period, with many of them being available for up to 30 months, as can be seen more precisely in the files in the “AAU_pilot_available_data_images” folder’s “Rooms” subfolder. In general, the room measurements are available for the period 2023–07–01 to 2024–07–01, with some lacking data, and many going further back.

### 3.3 NEST pilot

The NEST (Next Evolution in Sustainable Building Technologies) is a modular research and innovation building located in Dübendorf, Switzerland. It is operated by Empa and Eawag. NEST functions as a living lab and vertical energy district where novel construction technologies, materials, energy systems, and circular-economy concepts are developed, tested, and demonstrated under real-life conditions. Its modular structure allows research and industry partners to integrate and replace experimental units over time.

The NEST building currently includes 10+ innovative units, among them the following four units are included in this dataset in line with the scope of Horizon Europe HEATWISE project:

• **UMAR (Urban Mining and Recycling)**: A pioneering circular construction unit that applies urban mining principles. It uses reclaimed, reused, and recyclable materials, and is designed for disassembly to enable material recovery and closed-loop building cycles.
• **Sprint**: A flexible and rapidly reconfigurable office unit designed to test adaptable building systems. It enables fast spatial transformations and supports research on user-centered design, lightweight construction, and agile workspace concepts.
• **DFAB House**: Developed within the National Centre of Competence in Research (NCCR) Digital Fabrication, this unit showcases digitally fabricated building components, including robotic construction processes and 3D-printed structural elements, demonstrating how digital technologies can enhance precision, efficiency, and sustainability in construction.
• **HiLo (High-Performance Low-Emissions)**: A lightweight, resource-efficient unit featuring an innovative thin-shell roof structure and advanced building systems. HiLo demonstrates how computational design, digital fabrication, and smart energy concepts can significantly reduce material use and operational emissions while maintaining high architectural and structural performance.

**Fig. 6:**
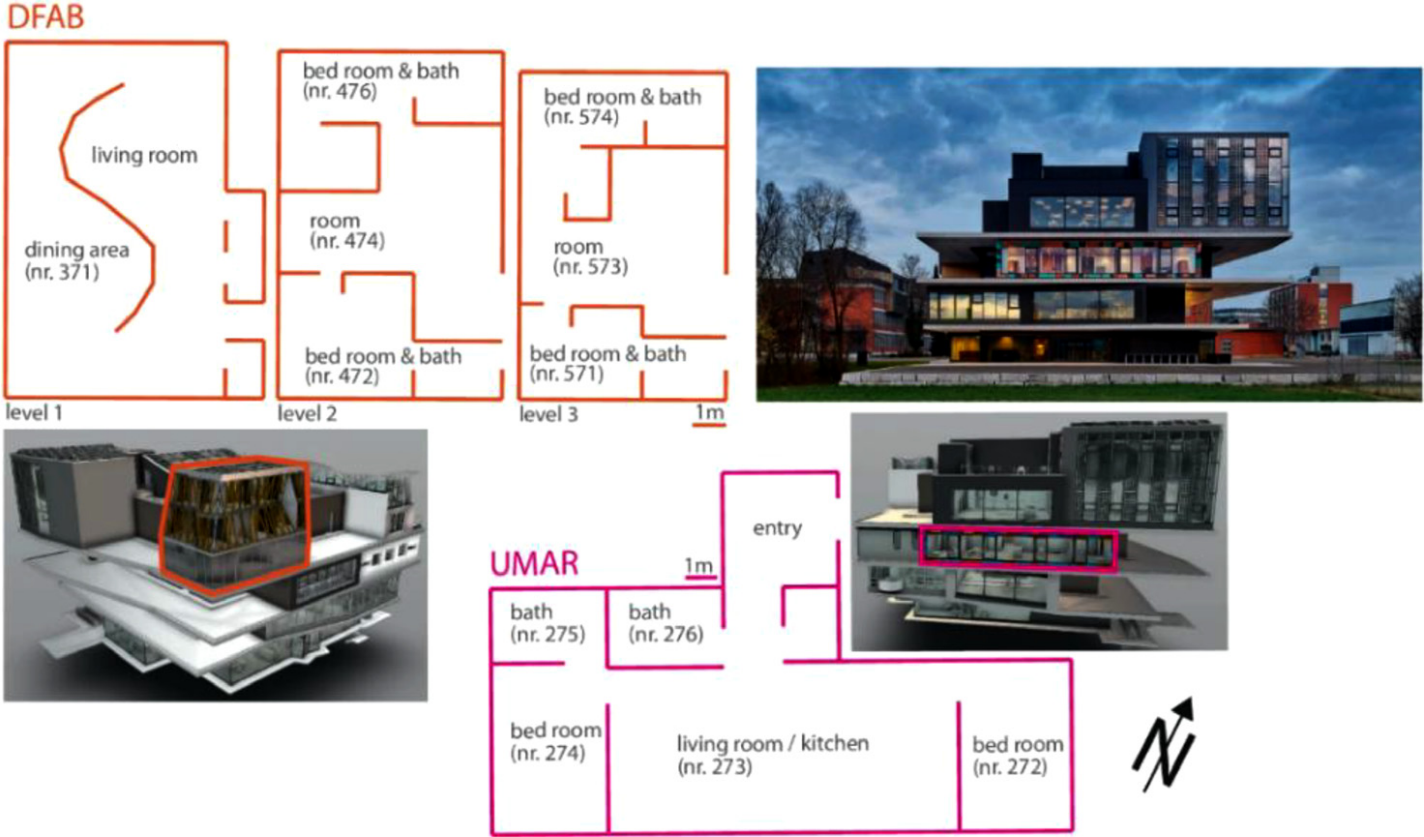
DFAB and UMAR unit at NEST, adapted from [5].

**Fig. 7:**
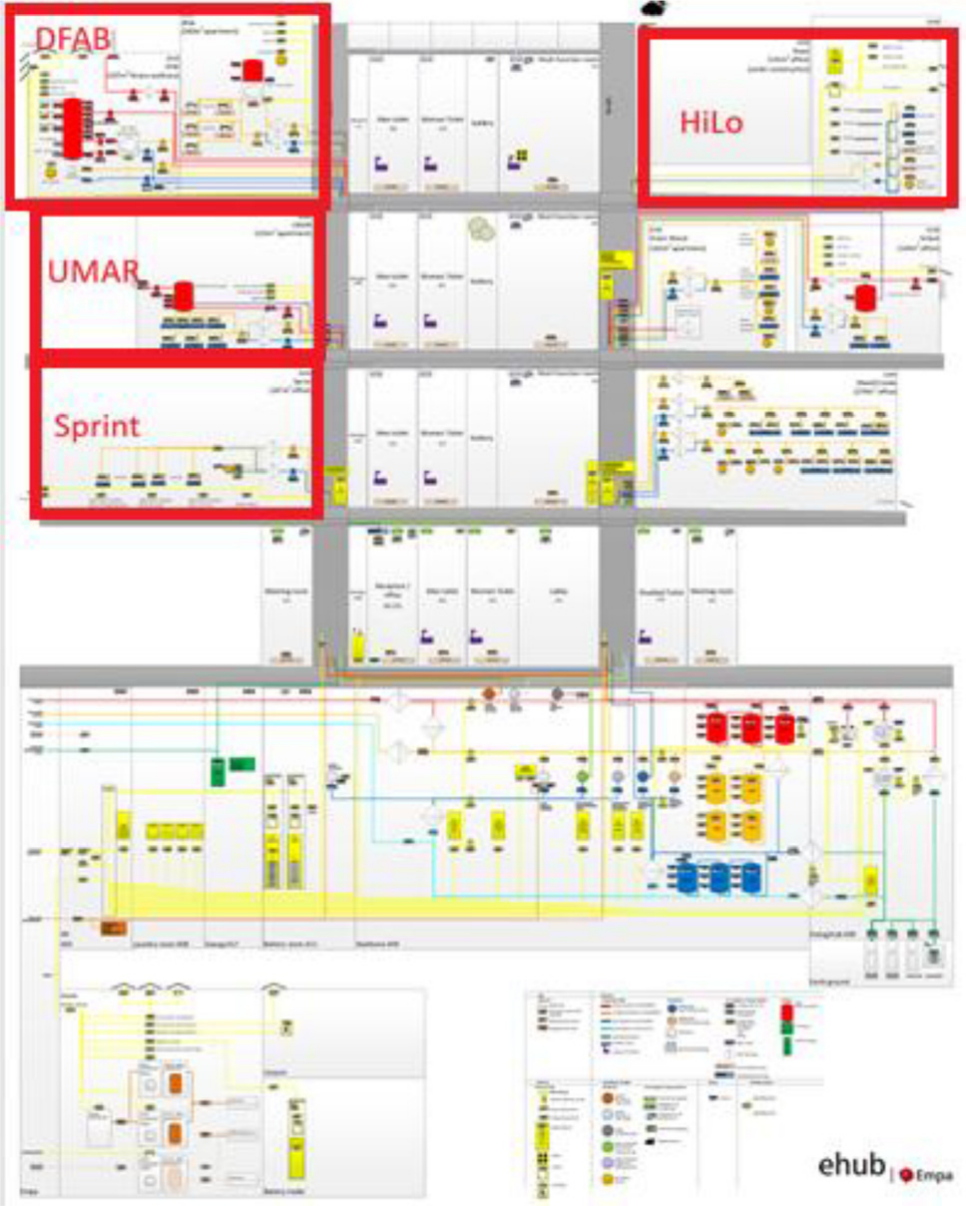
Overview and location of UMAR, Sprint, HiLo, and DFAB buildings at the NEST demonstrator (all are seen from the side).

Fig. 6 illustrates the location and details of DFAB and UMAR, both residential buildings at the NEST demonstrator, while Fig. 7**.** illustrates the location of all four units included in the NEST pilot building data set.

#### 3.3.1 NEST dataset

The NEST dataset consists of 19 individual data files (.csv format) with 333 data points, all described in the overview file called “data_overview.xlsx” in the “NEST_pilot” tab.

In the folder “NEST_pilot_images.zip”, the images are organized into the subfolders “Available data images”, “Floor Plans”, and “Schematics”. The subfolder "Available data images" contains images,showing the data availability for each unit and meter. The "Floor Plans" subfolder contains all floor plans for the four buildings units at NEST. The subfolder "Schematics" contains all the measurement concepts of the four buildings. The floor plans and schematics are included and described in this article, whereas the available data images can only be seen in the dataset.

For each building, the electricity meter, thermal heating meter, heating system, and room control data are available. Fresh water system data is only available for UMAR and DFAB building. The time resolution is 1 min, and the data is raw without preprocessing. A general overview of the 19 data files is presented in Table 3. It consists of four different buildings within NEST, as well as a weather station.

**Table 3:** Overview of NEST data files along with their category.

Type of Data	Building Umar	Building Sprint	Building Hilo	Building DFAB	Weather Station
**Electric Meter**	umar_electric_ meter	sprint_electric_ meter	hilo_electric_ meter	dfab_electric_ meter	
**Thermal Meter**	umar_thermal_ meter	sprint_thermal_ meter	hilo_thermal_ meter	dfab_thermal_ meter	
**Heating System**	umar_heating_ system	sprint_heating_ system	hilo_heating_ system	dfab_heating_ system	
**Fresh Water System**	umar_fresh_ water			dfab_fresh_ water	
**Room Control**	umar_room_ control	sprint_room_ control	hilo_room_ control	dfab_room_ control	
**Outdoor Data**					outdoor_weather_ station

For all the data of the NEST pilot, the timestamp is provided in the following format “YYYY-MM-DD hh:mm:ss+hh:mm” with the timezone corresponding to Swiss local time, which is either UTC+1 (CET) or UTC+2 (CEST). The data is generally available in the period from 2023 to 11–01 to 2024–08–01.

#### 3.3.2 Equipment identification system and symbol description

**Fig. 8:**
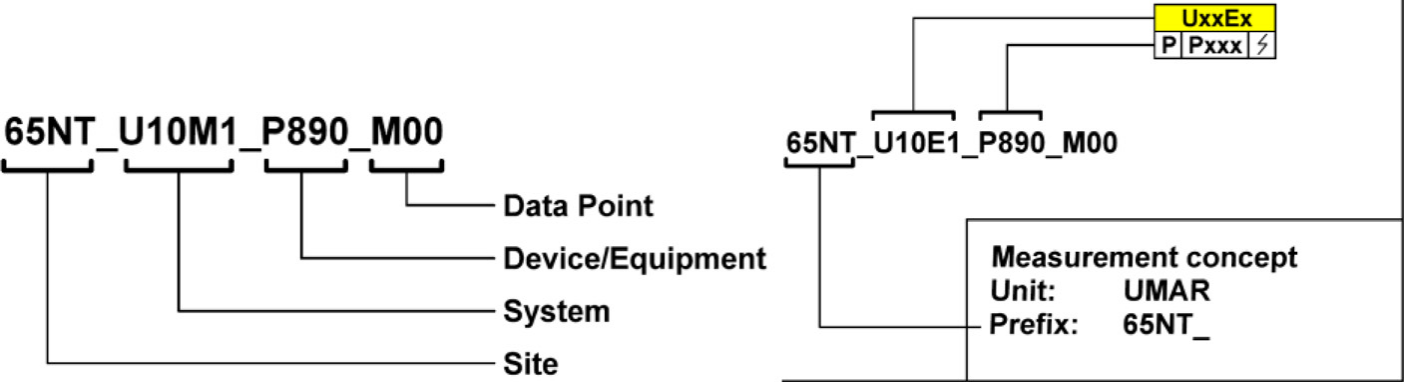
Equipment identification system (AKS) at the NEST demonstrator (right), and an example of this AKS (left).

The NEST demonstrator employs an equipment identification system (AKS) as illustrated in Fig. 8. The relationship between AKS and Unique ID in the variable name is provided in the “Data_Overview.xlsx” file in the “NEST_Pilot” tab.

**Fig. 9:**
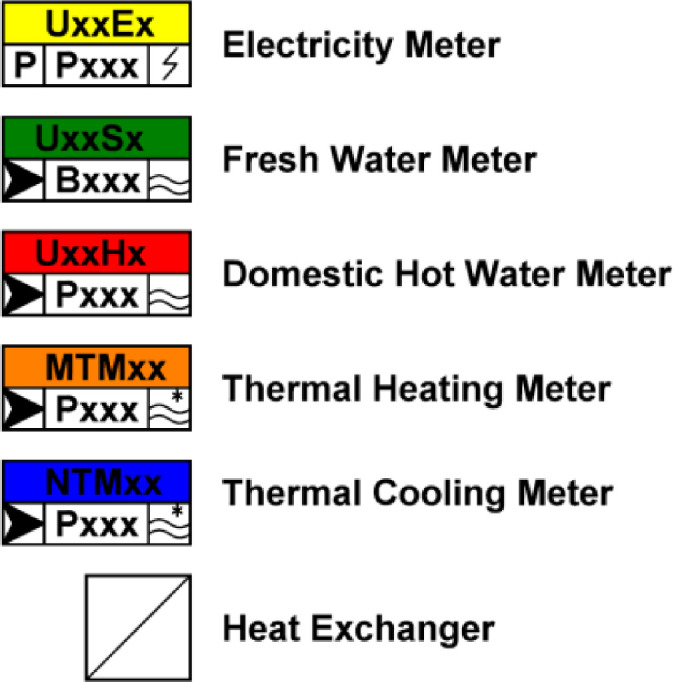
Symbols used in measurement concepts for NEST. The cooling meter data and domestic hot water data are not available in this dataset, but are only included here for completeness.

The symbols in Fig. 9 are used in the measurement concept schematics found in the “NEST_pilot_images.zip” folder, which describe the datapoints in files found in the “NEST_pilot_data.zip” folder where the naming is as follows (xx is the name of the building):

• xx_electric_meter
• xx_thermal_meter
• xx_fresh_water

**Electricity meter data**: The electricity meter measures electrical energy and power. The meter can have sub-meters, as presented in the schematics. Unmeasured consumers and producers within the building are also present. The data files provided in the “NEST_pilot_data.zip” folder contain the main meters of the building described.

**Fresh meter data:** The two buildings (UMAR, DFAB) have a fresh water supply. The meter measures water usage.

**Thermal heating meter data**: The thermal meter measures the water flow, the inlet- and outlet temperatures. Then they calculate the power and energy from the measurements. The meter can have sub-meters, which are visible in schematics.

**Heating system data:** The primary purpose of the heating system is to provide heating energy during the winter. The heat is sourced from the district heating network. The heating is separated from the district heating network by a heat exchanger. The heat is generated locally within the demonstrator using heat pumps or waste heat from data centers or imported from the Empa campus grid.

The system is switched on/off via adjustable heating limits. The system is operated via weather-compensated inlet flow temperature control. The set point for the inlet flow temperature is calculated as a function of the outdoor temperature via an adjustable heating curve.

The energy is dissipated via ceiling heating panels, the thermally activated building systems (TABS) system, or the floor heating system. The heating system also provides energy for the Air handling unit.

Individual room control is implemented in the buildings. Each thermal zone or room measures the room temperature, and the thermostat then controls the corresponding regulating valves. The heating system file stores the regulating valve data.

**Room control**: Various room sensors are installed in the buildings, along with thermostats that have a set temperature point. In the corresponding schematics, the sensor location and type are clearly visible.

#### 3.3.3 UMAR

**Fig. 10:**
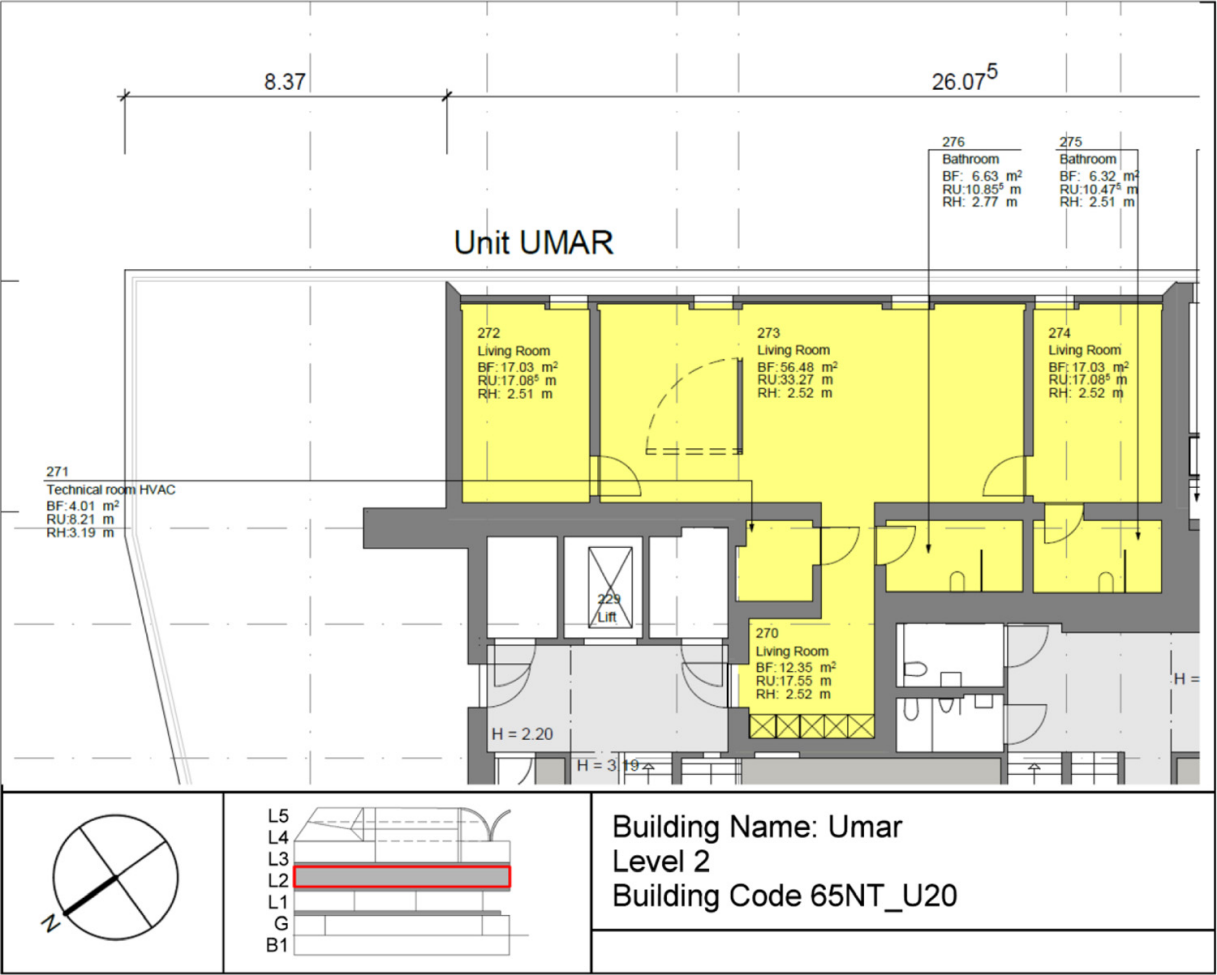
Floor plan UMAR building.

The Urban Mining & Recycling (UMAR) building is located on the second floor of the NEST demonstrator as illustrated in Fig. 6 and Fig. 7. It has been operational since 8th of February 2018, and demonstrates how a sustainable use of natural resources can be aligned with appealing architecture. It is a single-storey residential building comprising two bedrooms, one common living room with a kitchen, and two bathrooms, with a total floor area of 155 m². A plan drawing of the building is shown in Fig. 10**.**

**Fig. 11:**
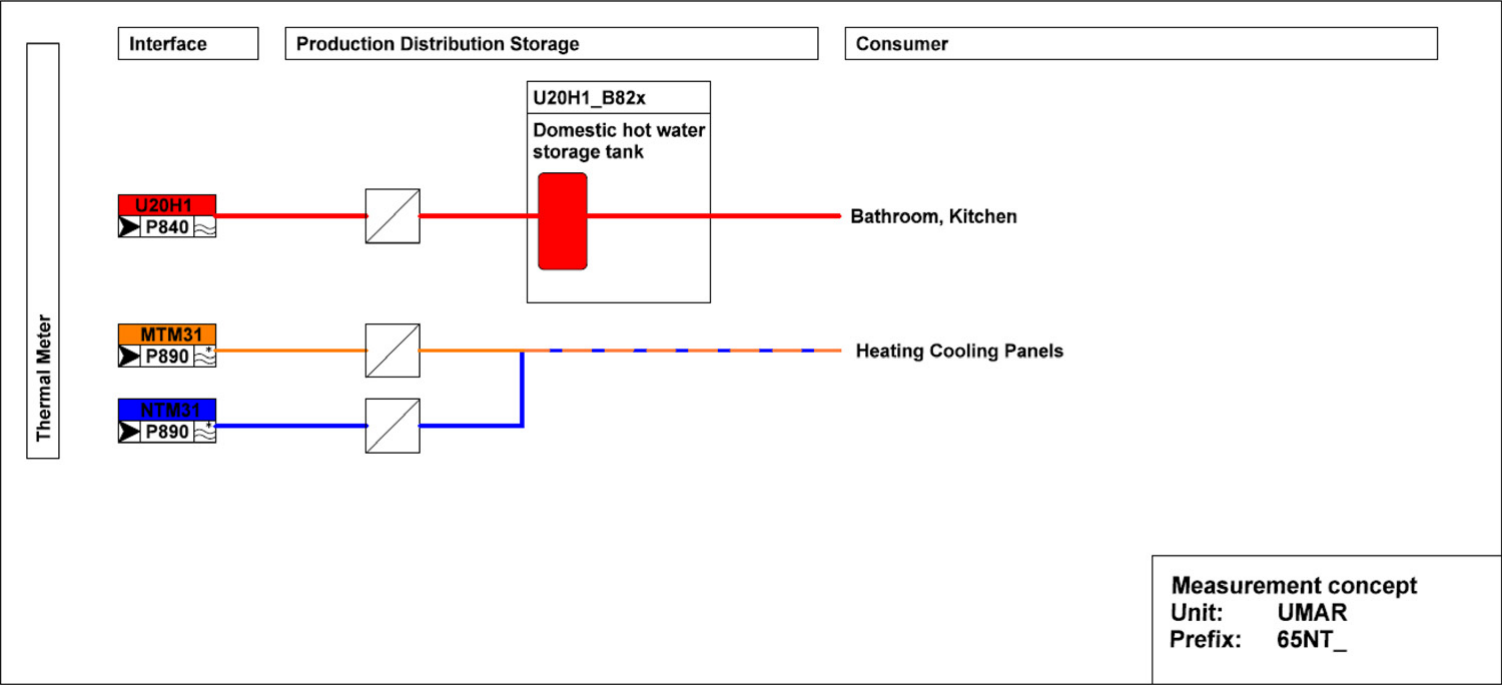
Measurement concept for thermal energy meter in UMAR building.

The measurement concepts for the thermal energy meter in UMAR are illustrated in Fig. 11. The main meter is located on the district heating connection to the building and separated from the district heating network by a heat exchanger. The thermal meter measures the water flow, the inlet- and outlet temperatures. Heating energy and power are derived from these measurements.

**Fig. 12:**
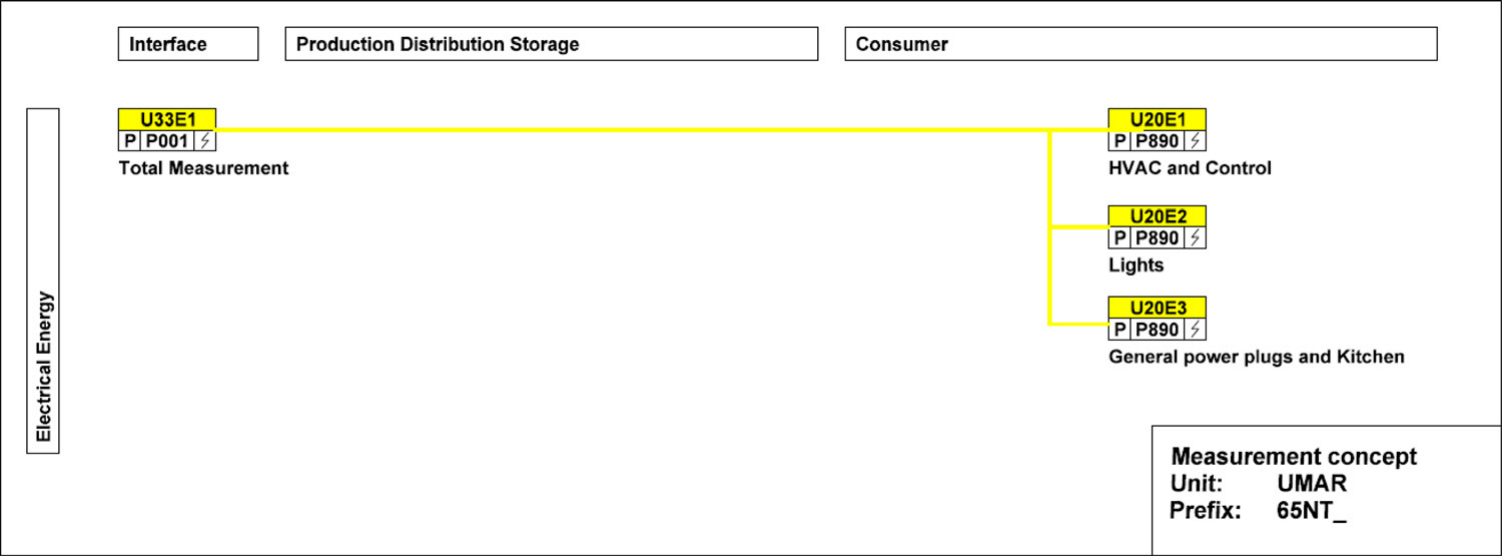
Electricity main meter and sub-meters distribution at UMAR.

Fig. 12 illustrates the main electricity meter that measures electrical energy and power to the UMAR building. It also has several sub-meters that cover lighting, general consumer/plug loads, kitchen, heating, ventilation, and air-conditioning (HVAC), and building automation.

**Fig. 13:**
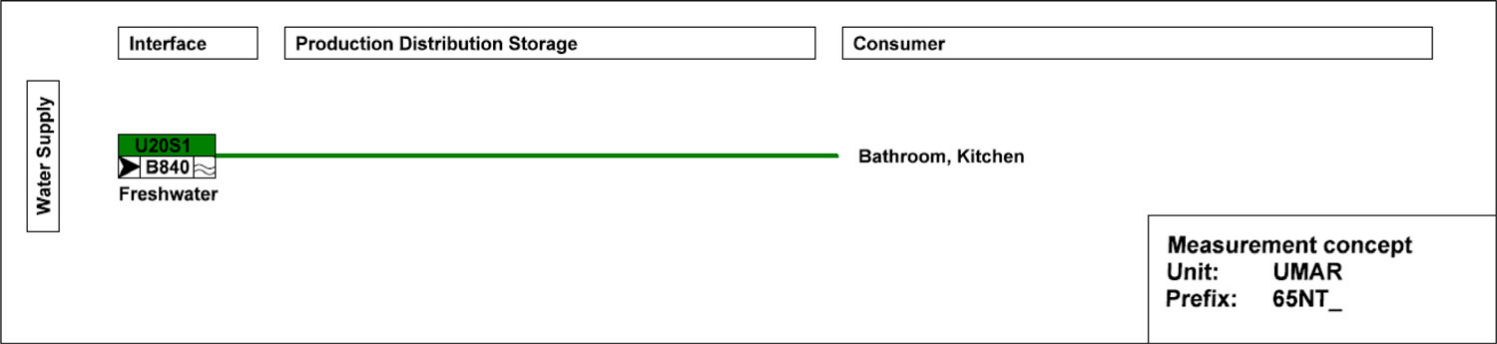
Measurement concept for fresh water in the UMAR building.

Fig. 13 shows the freshwater connection for the bathroom and kitchen. It measures water usage in the building.

The availability of data from the electricity meter, thermal meter, heating system, room control, and fresh water in the UMAR building is illustrated in the “umar_xx.pdf” images in the “NEST_pilot_images” folder’s “Available data images” subfolder. In general, no significant periods of data are missing.

#### 3.3.4 Sprint

**Fig. 14:**
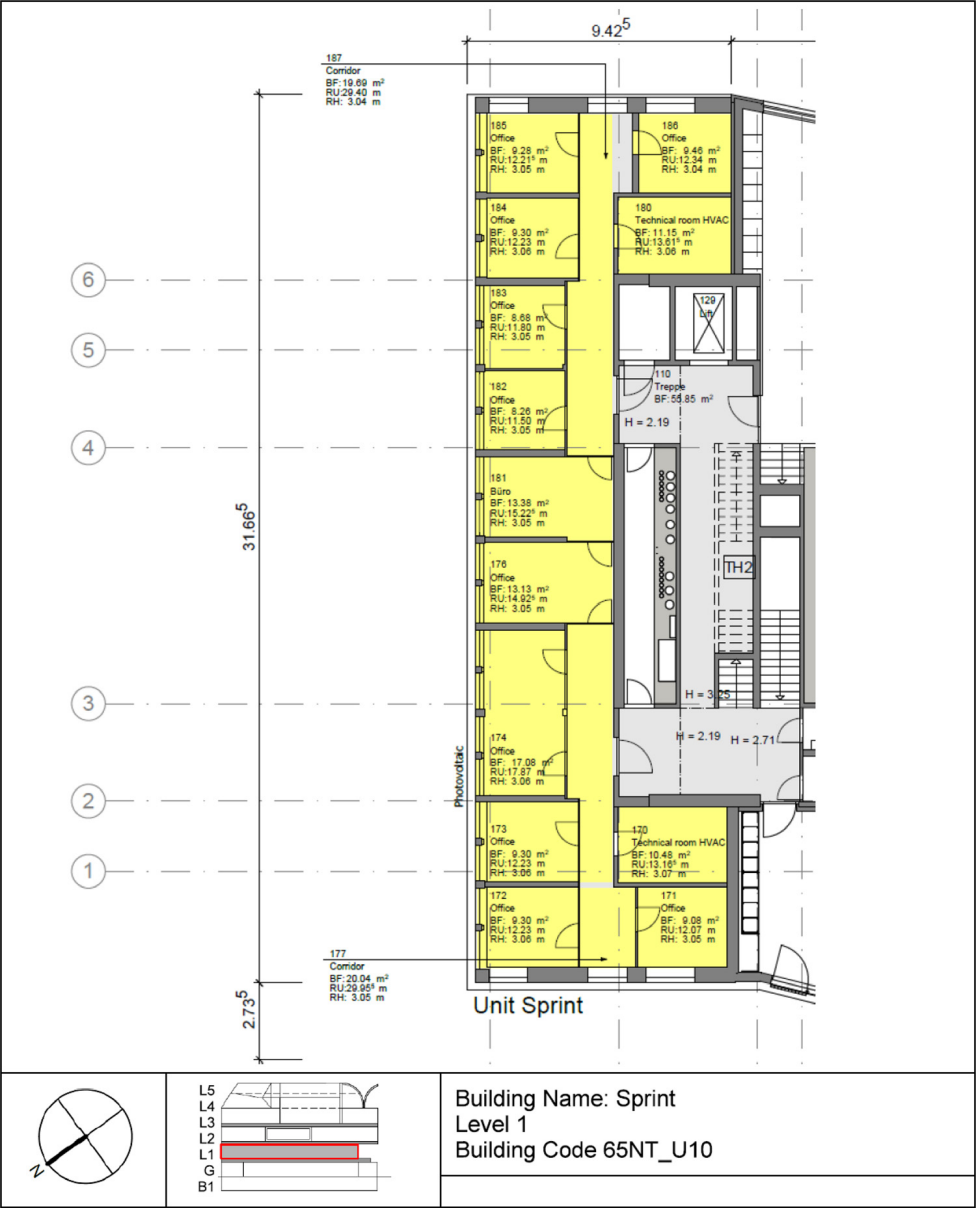
Floor plan Sprint building.

The Sprint building is located on the first floor of the NEST demonstrator, as illustrated in Fig. 7. It opened on the 16th of August 2021, and demonstrates the construction of flexible office space (to cover individual office space demand post Covid-19) within the shortest possible time using largely reused materials. It is a single-story office building designed for 12 people, featuring 16 rooms with a total floor area of 167 m². The floor plan is reported in Fig. 14.

**Fig. 15:**
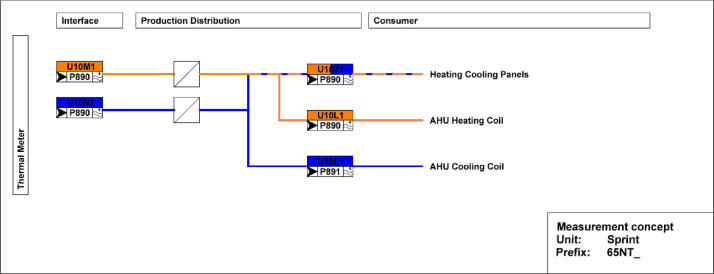
Measurement concept for thermal meters in the Sprint building.

Fig. 15 illustrates measurement concepts for thermal meters in the Sprint building. The main meter is located on the district heating connection to the building and separated from the district heating network by a heat exchanger. The thermal meter measures both the water flow and the temperatures at the inlet and outlet. The heating energy and power are derived from these measurements. There are submeters to measure the consumption of heating and cooling panels as well as air handling units.

**Fig. 16:**
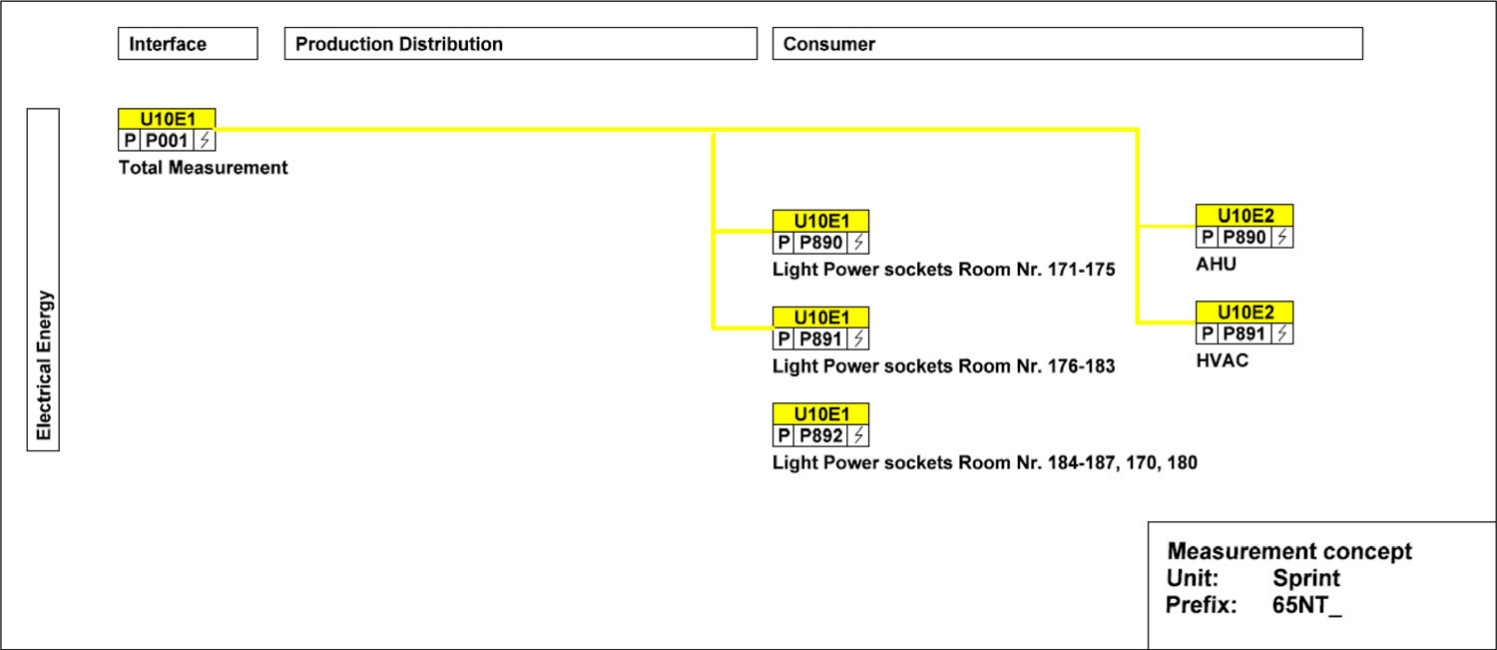
Electricity distribution of the Sprint Unit.

The main electricity meter and the sub-meters for the different end-uses and areas are illustrated in Fig. 16. The main electricity meter measures electrical energy and power to the Sprint building. It also features several sub-meters that cover lighting and power sockets in different areas within the building, as well as an air-handling unit and HVAC systems.

The availability of data from the electricity meter, thermal meter, heating system, and room control in the Sprint building is illustrated in the “sprint_xx.pdf” images in the “NEST_pilot_images” folder’s “Available data images” subfolder. In general, no significant periods of data are missing.

§

#### 3.3.5 DFAB

**Fig. 17:**
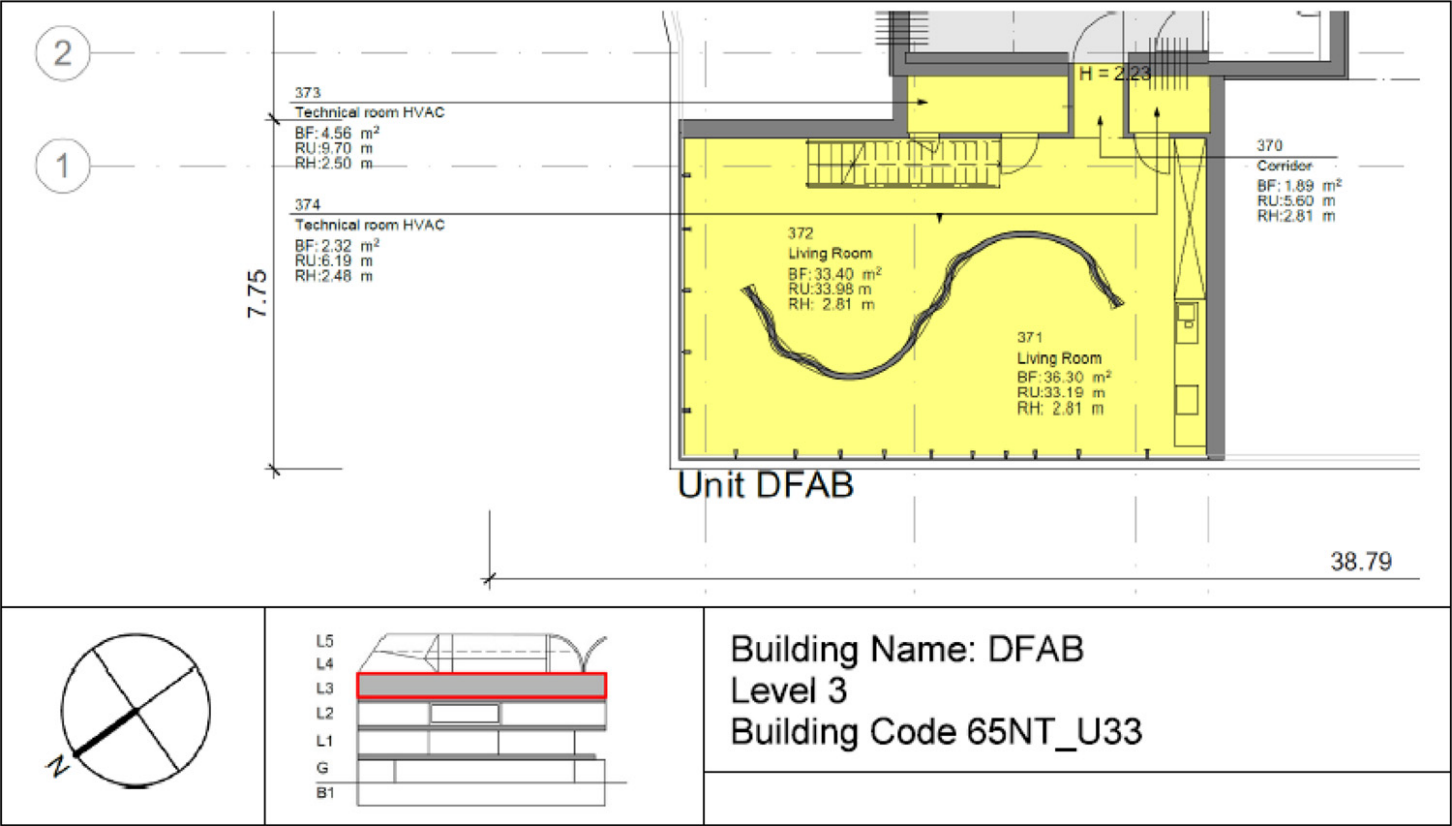
DFAB building 1st floor.

**Fig. 18:**
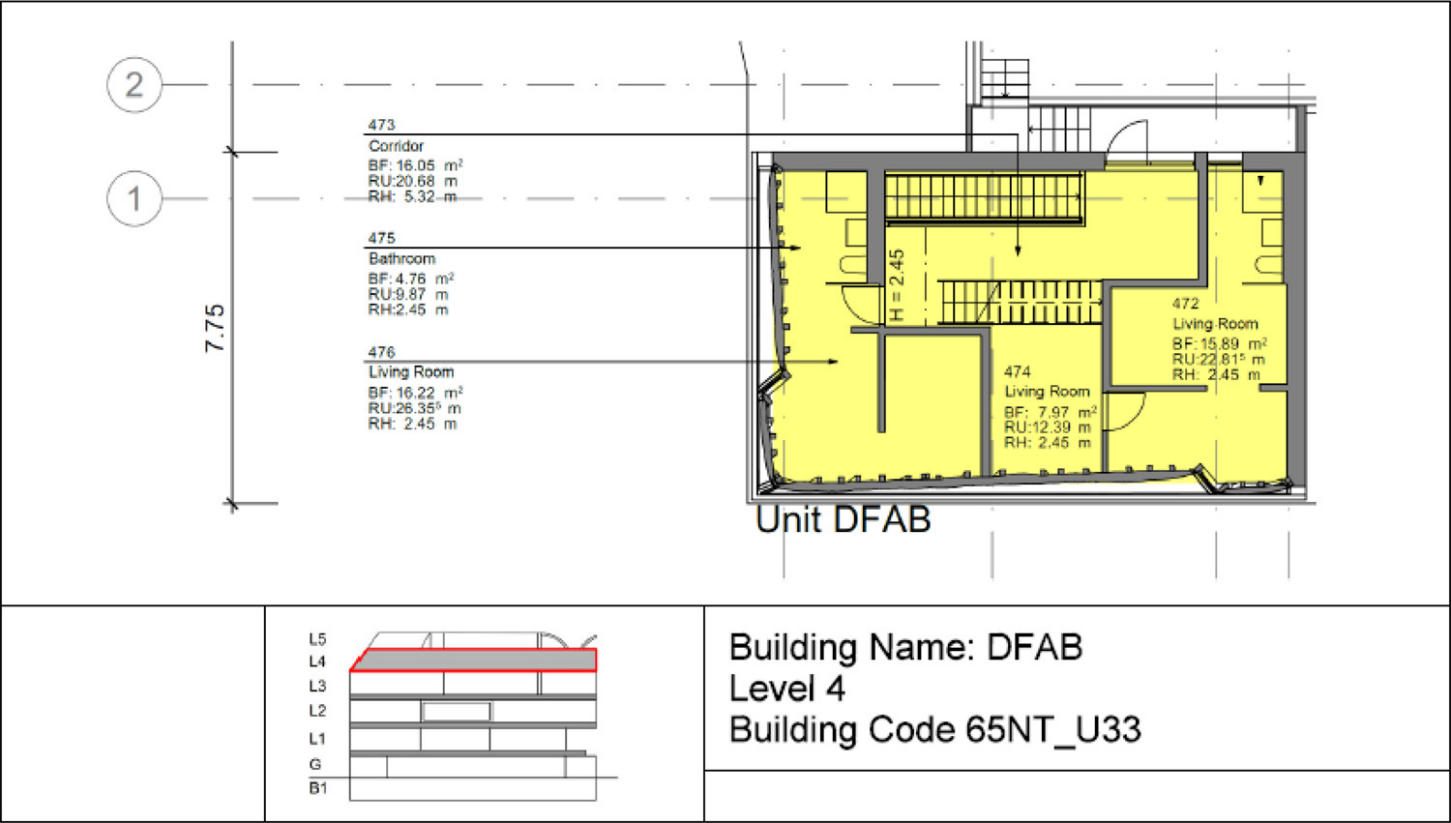
DFAB building 2nd floor.

**Fig. 19:**
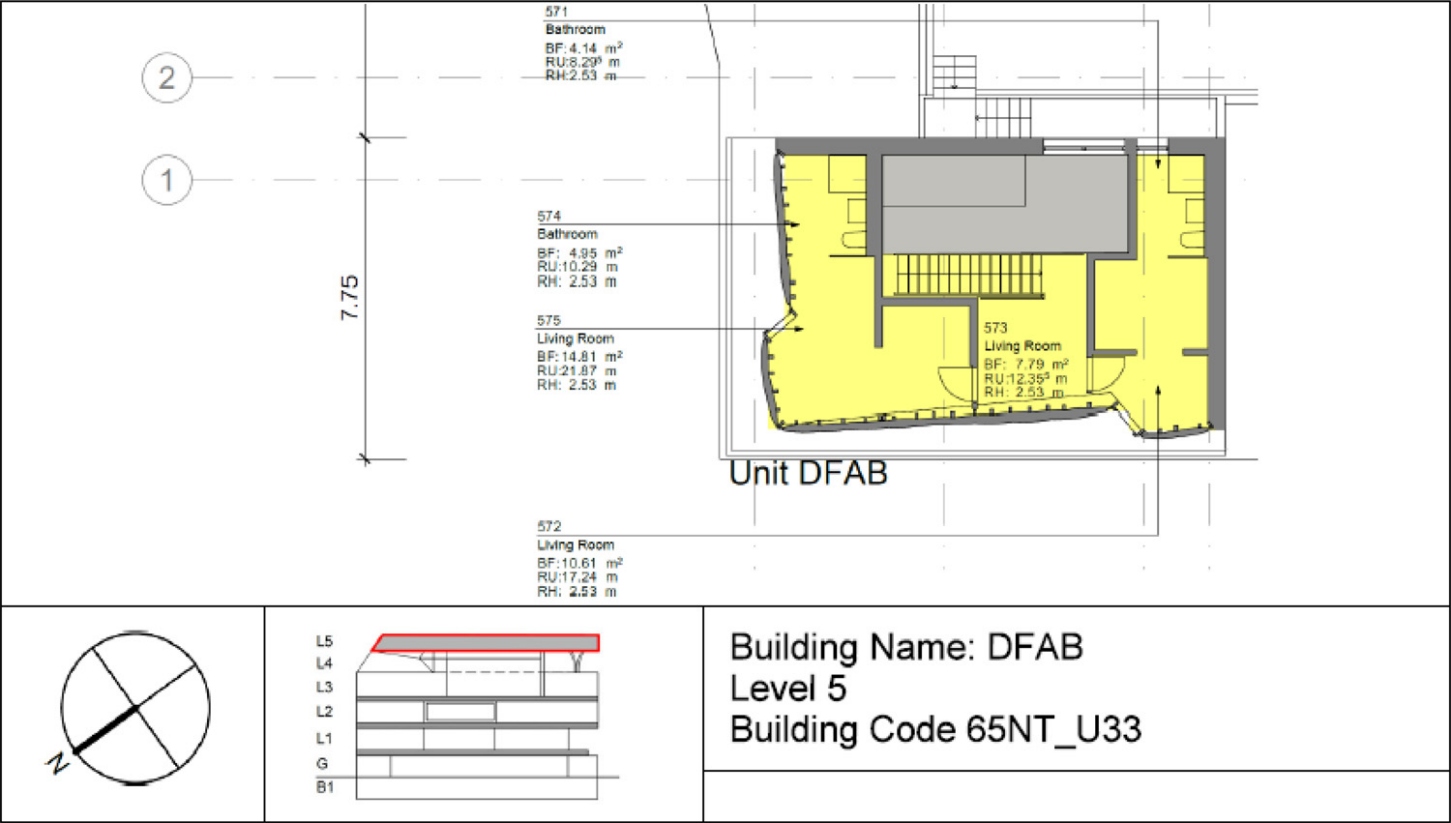
DFAB building 3rd floor.

The DFAB building is located on the third floor of the NEST demonstrator, as illustrated in Fig. 6, Fig. 7. It opened in February 2019 and is the world’s first residential building that was not only digitally planned but also predominantly digitally built using robots and 3D printers. The DFAB is a residential building with three floors and three bedrooms (Fig. 17, Fig. 18, and Fig. 19). The heating system is designed to provide thermal energy during winter. The building is connected to the medium temperature (MTE) grid via a heat exchanger. Heat is distributed through a floor heating system. Temperature settings for each heating circuit group are individually controlled through room thermostats. Additionally, a ventilation system has been installed. An on-site heat pump and boiler, combined with photovoltaic panels, cover the domestic hot water demand. There is no connection to the high temperature (HTE) grid.

**Fig. 20:**
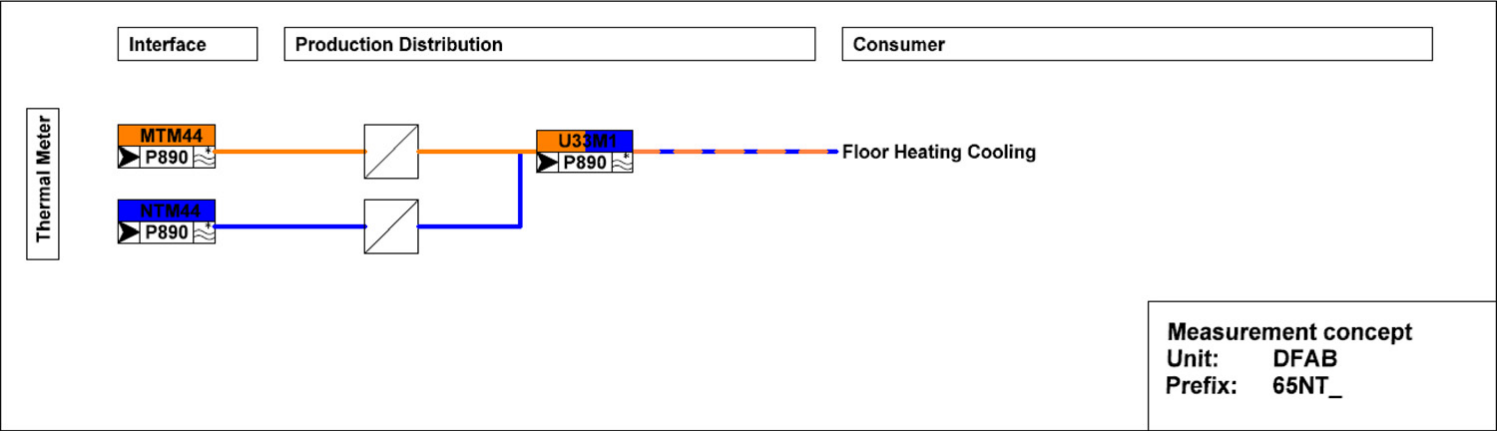
Measurement concepts for thermal meters in the DFAB building.

Fig. 20 shows measurement concepts for thermal meters in the DFAB building. The main thermal meter is located on the district heating connection to the building and separated from the district heating network by a heat exchanger. The thermal meter measures both the water flow and the temperatures at the inlet and outlet. The heating energy and power are derived from these measurements. There are submeters to measure consumption in floor heating and cooling systems.

**Fig. 21:**
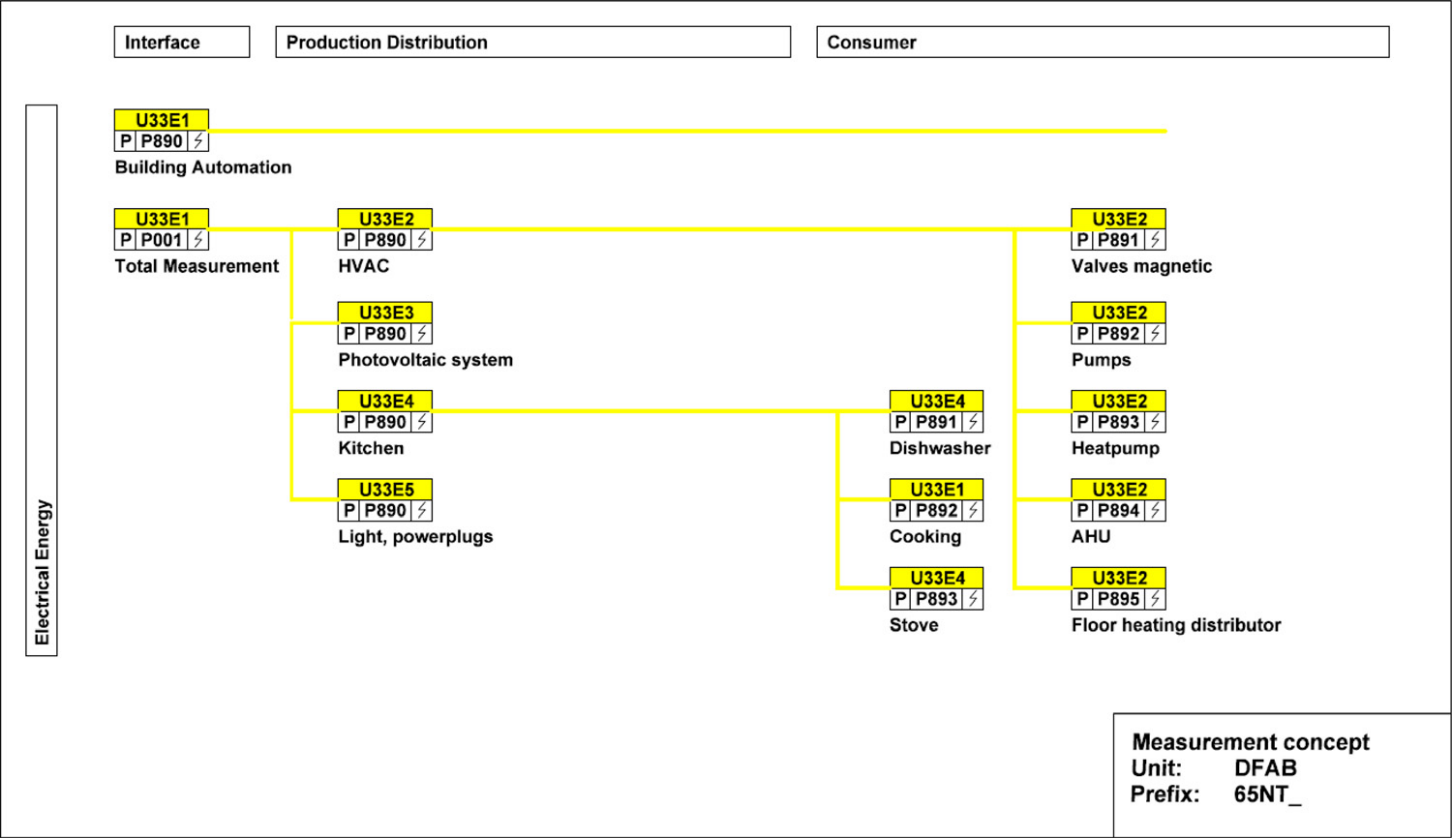
Electricity distribution in the DFAB Unit features separate metering for the kitchen and HVAC-related systems.

In Fig. 21, the main electricity meter and the sub-meters for the different end-uses are illustrated. The main electricity meter measures electrical energy and power to the DFAB building. Its sub-meters cover lighting and power sockets, the kitchen, photovoltaics (PV) systems, as well as HVAC systems. The kitchen features additional sub-meters to measure consumption, including those for the dishwasher, cooking, and stove. Similarly, HVAC also includes additional sub-meters to measure consumption of magnetic valves, pumps, heat pumps, air handling units, and floor heating distributors.

**Fig. 22:**
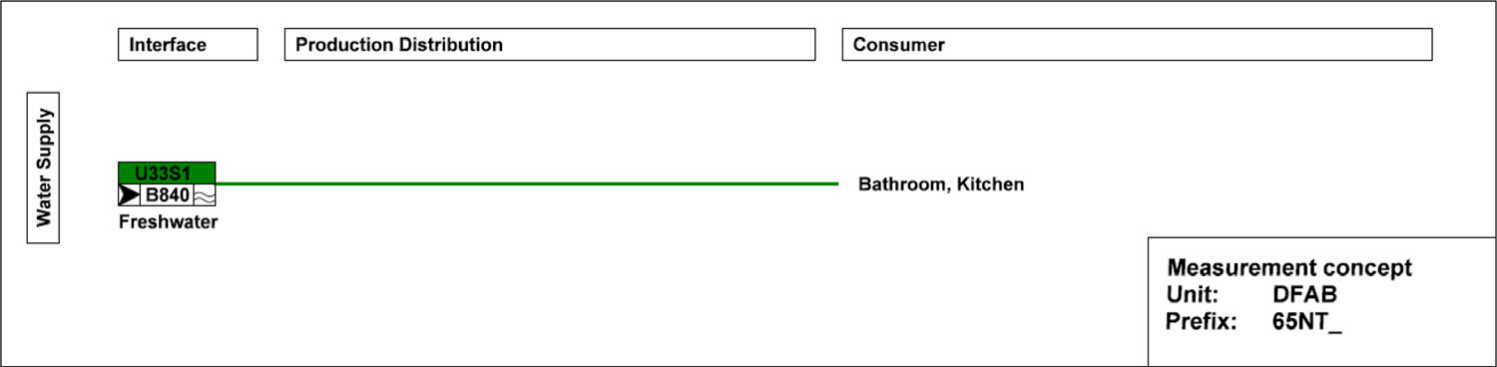
Measurement concept for fresh water in the DFAB building.

Fig. 22 shows the freshwater connection for the bathroom and kitchen of the DFAB building. It measures water usage in the building.

The availability of data from the electricity meter, thermal meter, heating system, room control, and fresh water in the DFAB building is illustrated in the “dfab_xx.pdf” images in the “NEST_pilot_images” folder’s “Available data images” subfolder. In general, no significant periods of data are missing.

#### 3.3.6 HiLo

**Fig. 23:**
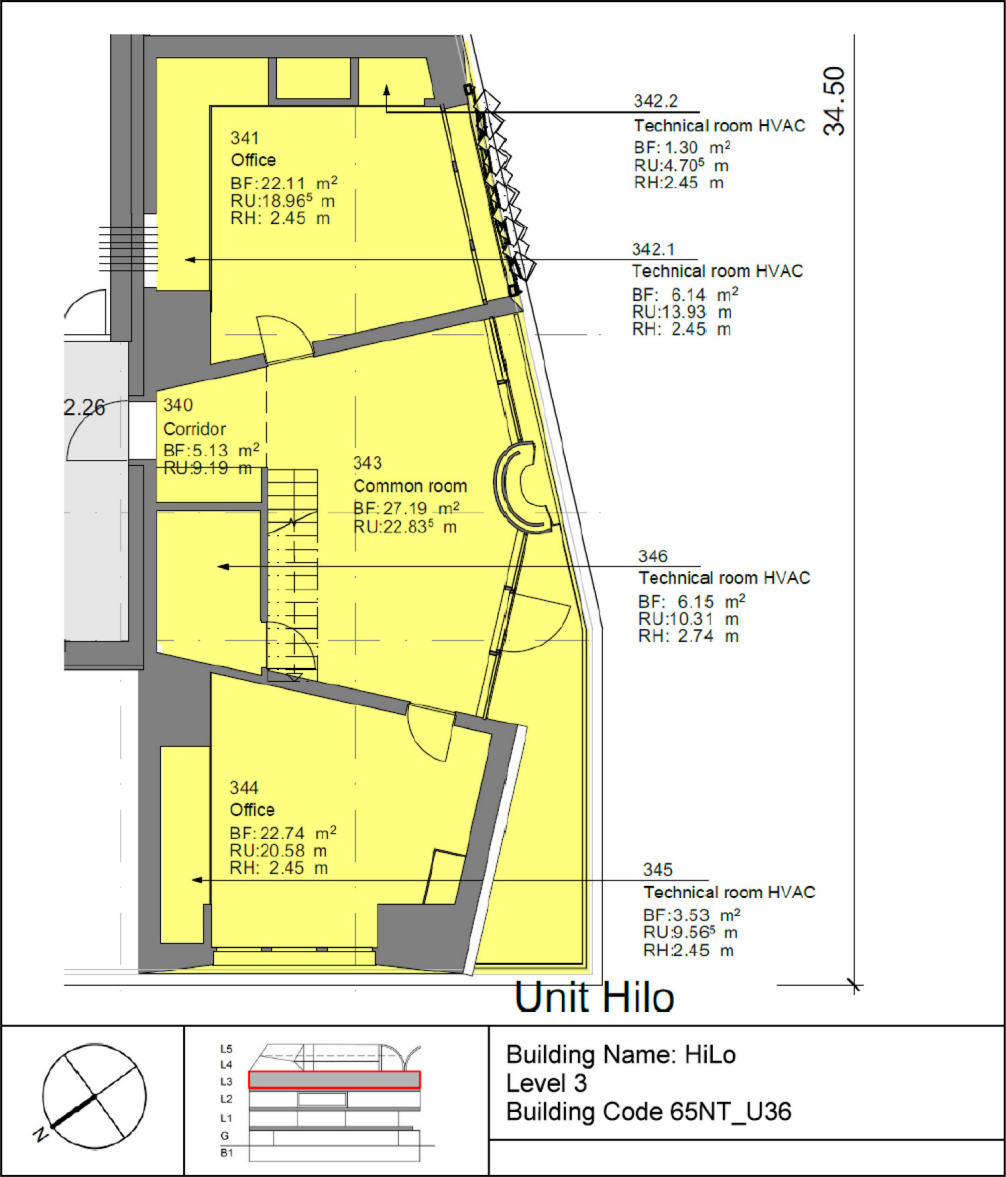
HiLo building 1st floor.

**Fig. 24:**
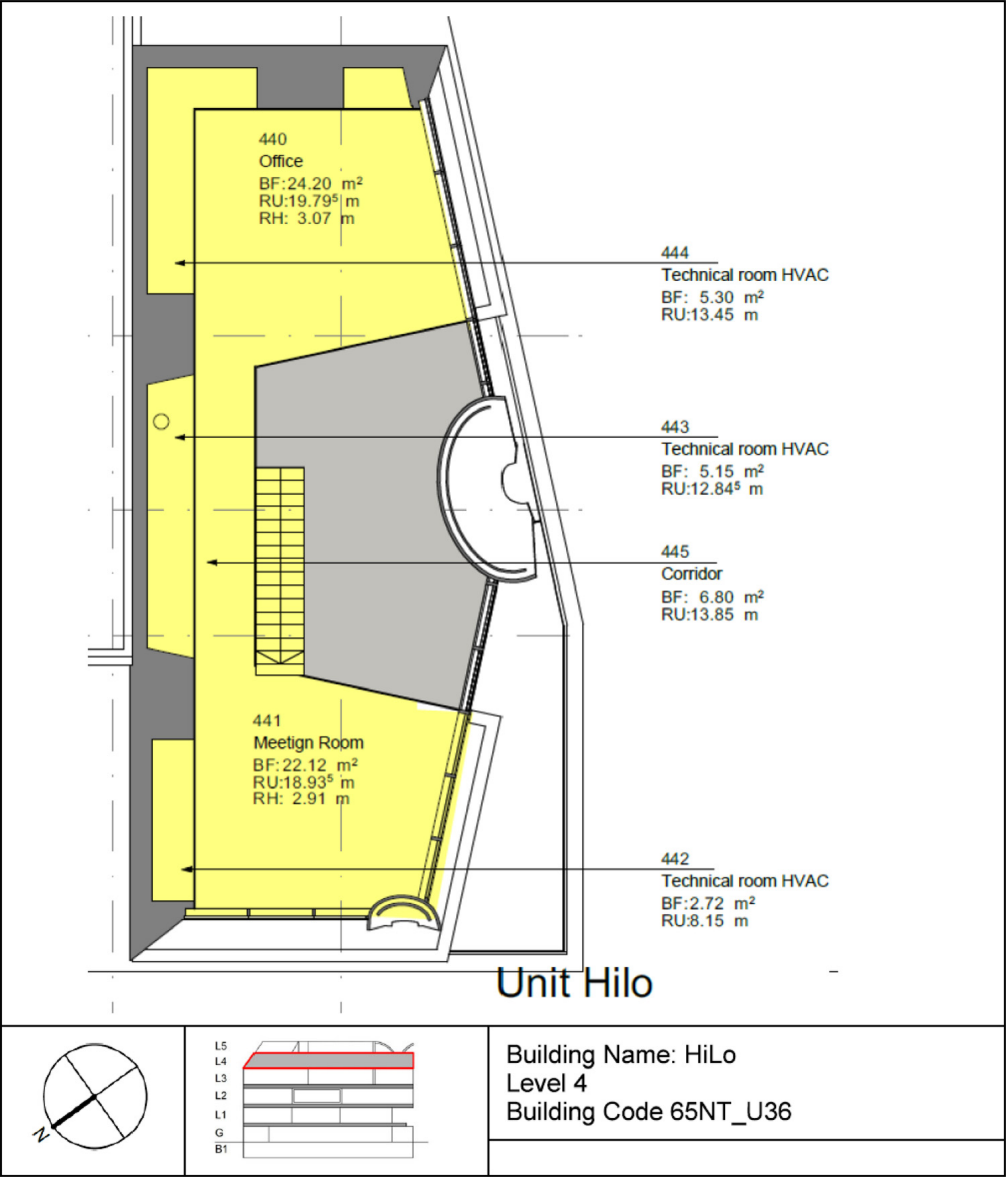
HiLo building 2nd floor.

The High Performance - Low Emissions (HiLo) building is located on the third floor of the NEST demonstrator, as illustrated in Fig. 7**.** It opened on the 28th of January 2022, and demonstrates how attractive architecture can be combined with energy- and resource-saving construction and operation. The HiLo building is a two-story office space for 8 people. It has 7 rooms with a total floor area of 159 m^2^. The floor plans of the building can be seen in Fig. 23 and Fig. 24.

**Fig. 25:**
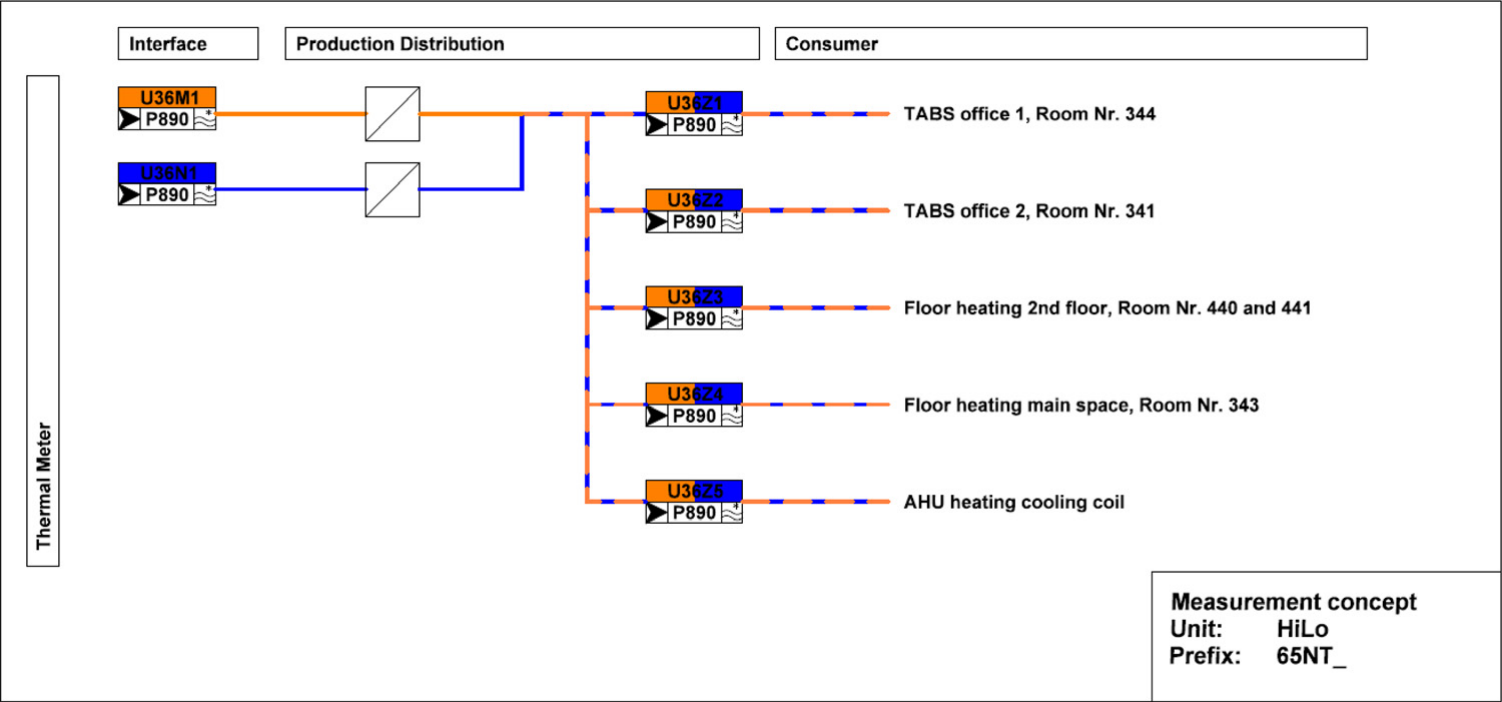
Measurement concepts for thermal meters in the HiLO building.

Fig. 25 shows measurement concepts for thermal meters in the HiLo building. The main meter is located on the district heating connection to the building and separated from the district heating network by a heat exchanger. The thermal meter measures both the water flow and the temperatures at the inlet and outlet. The heating energy and power are derived from these measurements. There are sub-meters to measure consumption in the TABS in rooms (341 and 344), floor heating in rooms (343, 440, and 441), as well as the heating and cooling coils of the air handling unit.

**Fig. 26:**
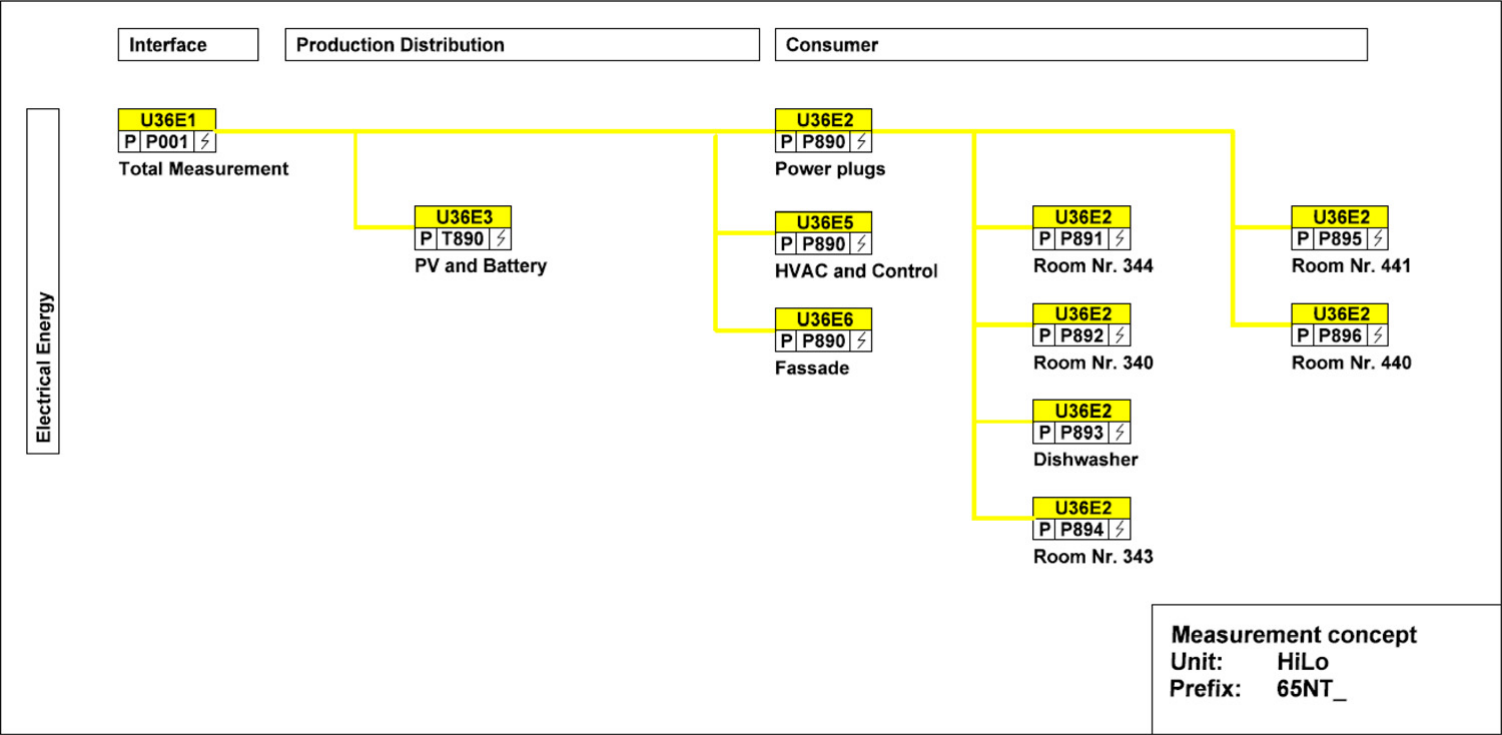
Measurement concepts for electrical energy meters in HiLo unit.

The main electricity meter and the sub-meters for the different end-uses are illustrated in Fig. 26. The main electricity meter measures electrical energy and power to the HiLo building. Its sub-meters cover PV and battery systems, façade, power sockets, as well as HVAC systems and building control. There are additional sub-meters to measure consumption in the power plugs of all the rooms and the dishwasher.

The availability of data from the electricity meter, thermal meter, heating system, and room control in the HiLO building is illustrated in the “hilo_xx.pdf” images in the “NEST_pilot_images” folder’s “Available data images” subfolder. In general, no significant periods of data are missing.

#### 3.3.7 Weather station

NEST is equipped with a weather station that provides access to outdoor weather measurements. The available measurements include outdoor air temperature, relative humidity, air pressure, wind speed, wind direction, and solar radiation. Additionally, the solar altitude azimuth, and solar altitude elevation are recorded. The forecast from MeteoSuisse [6] is also available. Forecasts are available for outdoor ambient temperature, solar irradiance, and outdoor relative humidity. The forecast is updated 4 times daily and predicts the next 72 h.

## 4 Experimental Design, Materials and Methods

### 4.1 AAU pilot

The measurements presented come directly from the Schneider EcoStruxure building management system (BMS) of the building. They are generally raw data, with the only pre-treatment being that the data points are aligned to their next minute, meaning that a data point measured at 12:41:52 becomes 12:42:00.

The AAU pilot building itself is designed and built according to the Danish building regulation BR class 2015, with a designed primary energy use of 56.3 kWh/m² per year. This corresponds to an energy performance certificate (EPC) label of A, and the EPC can be found here [7]. For more general information regarding the building, see the report by H. Johra [8].

#### 4.1.1 Main meters

The main meters generally have two separate measurement types, either cumulative (energy use and volume) or instantaneous (power and flow). If possible and available, it is encouraged to use the cumulative measurements, as these sum correctly over time even in periods of missing data. The instantaneous measurements are on the other hand, as instantaneous implies, power or flow measurements taken at a specific time, which in the case of highly fluctuating systems can cause the measurements to not be representative of the general tendency of the system. This is generally true if the measurements are taken and randomly hit all the high peaks of the fluctuations.

#### 4.1.2 Space heating

**Fig. 27:**
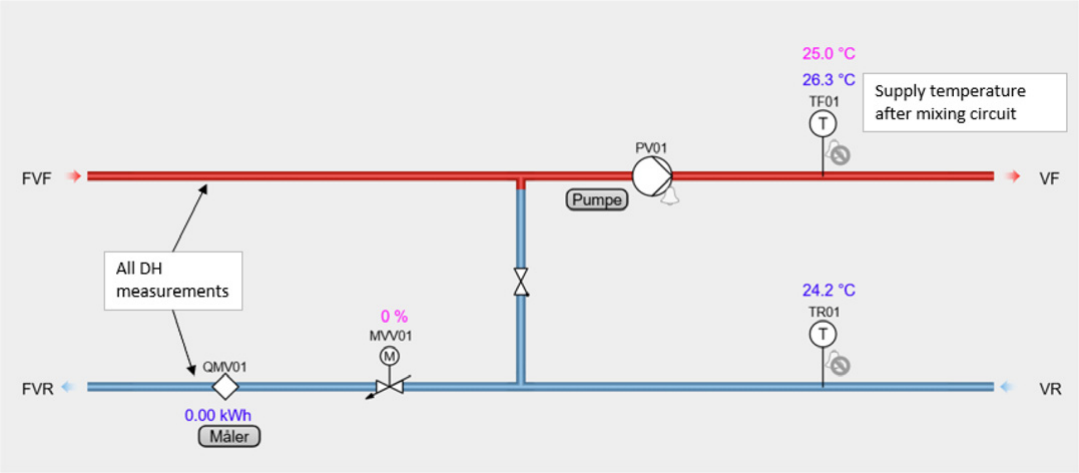
Space heating schematic.

The space heating system in the building is mainly a convective radiator-based system, with a few floor heating areas. For all of the individual circuits, there is a coupling to the main DH supply, which is then fed into a mixing loop, to be able to maintain the desired supply temperature, as seen in Fig. 27. The circulation pumps for the convective radiator circuits operate according to a constant speed setting, while those for the floor heating circuits operate with constant flow.

#### 4.1.3 Domestic hot water

**Fig. 28:**
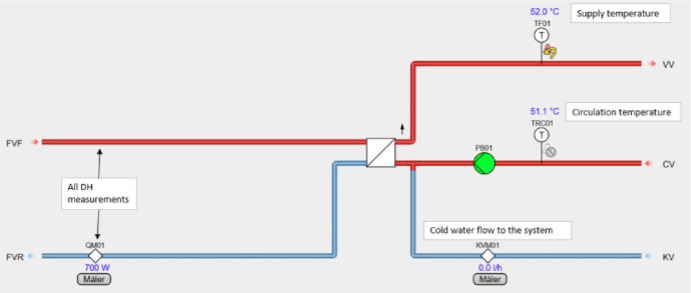
DHW system schematic.

All the different DHW circuits operate with the left side being the DH supply side with a direct connection, while the DHW side is separated from it by a heat exchanger. The DHW is circulated in the circuit, with cold water being added when water is drawn from the system. This is all heated up by the heat exchanger trying to maintain the proper water temperature. The schematic of the system can be seen in Fig. 28.

#### 4.1.4 Ventilation

**Fig. 29:**
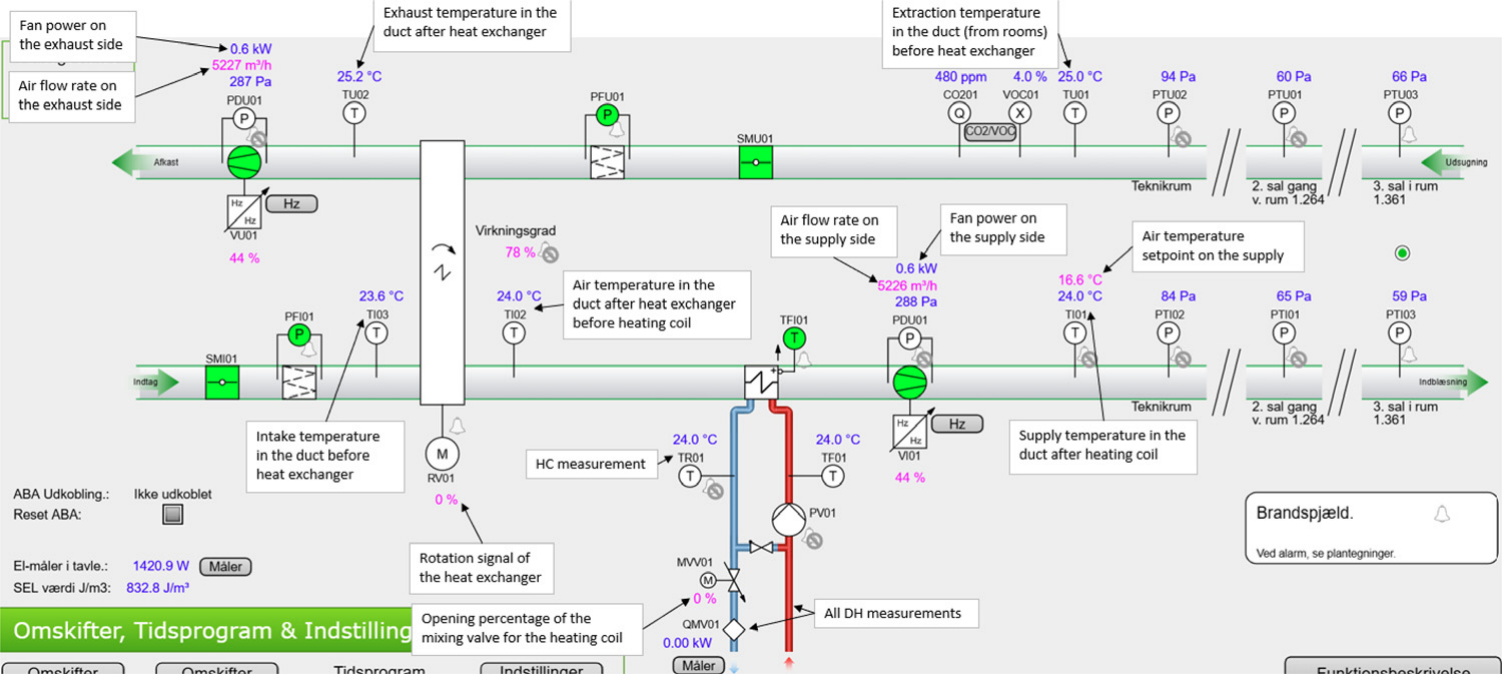
KOMF01 schematic.

**Fig. 30:**
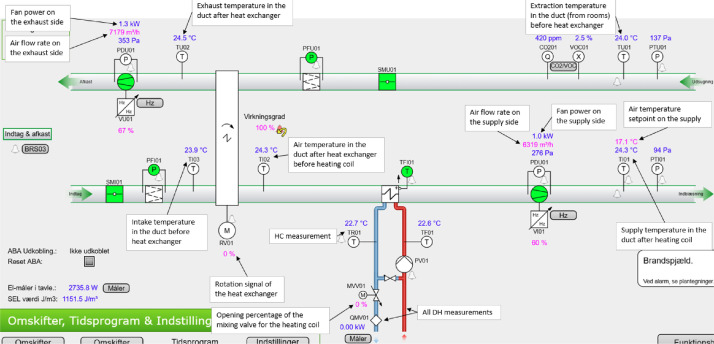
KOMF02 schematic.

For the ventilation units “KOMF01” and “KOMF02”, they have the full set of measurements; and the location of the different measurement points is therefore shown in Fig. 29 and Fig. 30. Both units are AHUs with an enthalpy heat recovery wheel and a water-based heating coil on the supply side. The main design values for both of the AHUs can be seen in Table 4.

**Table 4:** KOMF01 and KOMF02 overview of specifications for components. *indicates the values calculated according to ASHRAE Standard 84 and ARI Standard 1060. The table is originally from [9] and modified with the permission of the authors.

	KOMF01		KOMF02	
	Supply side	Exhaust side	Supply side	Exhaust side
Flow [m³/h]	10,000	10,000	12,000	12,000
Total pressure loss [Pa]	400	386	402	380
Fan manufacturer and model	Ziehl-Abegg ER56C-4DN.E7.CR	Ziehl-Abegg ER56C-4DN.E7.CR	Ziehl-Abegg ER63C-6DN.G7.CR	Ziehl-Abegg ER63C-6DN.G7.CR
Fan max rotational speed [RPM]	1242	1232	1084	1070
Fan K-factor	308	308	381	381
Fan motor manufacturer and model	Ziehl-Abegg IEC BG 100 L −4 m. termistor	Ziehl-Abegg IEC BG 100 L −4 m. termistor	Ziehl-Abegg IEC BG 132S −6 m. termistor	Ziehl-Abegg IEC BG 132S −6 m. termistor
Fan motor rated power [kW]	2.2	2.2	3.0	3.0
Fan motor max rotational speed [RPM]	1440	1440	960	960
HC manufacturer and model	ttc HW-ET-3,0–1480–1140–1R-6-V2-Cu/Al		ttc HW-ET-2,0–1480–1140–1R-6-V1-Cu/Al	
HC capacity [kW]	27.00		34.00	
Rotary HX manufacturer and model	Eventus ST1-SL-W-2265-CS-C1		Eventus ST1-SL-W-2480-CS-A1	
Rotary HX temperature effectiveness* [%]	86.0		86.0	
Rotary HX humidity effectiveness* [%]	66.7		66.7	
Rotary HX total effectiveness* [%]	82.7		82.7	
Rotary HX max rotational speed [RPM]	12		12	
Rotary HX motor power [W]	150		90	
Rotary HX total heat recovery capacity [kW]	116.7		140.0	
Rotary HX sensible heat recovery capacity [kW]	101.3		121.5	
Rotary HX latent heat recovery capacity [kW]	15.4		18.5	
Mass transfer of humidity [kg/h]	22		26	

#### 4.1.5 Miscellaneous

**Fig. 31:**
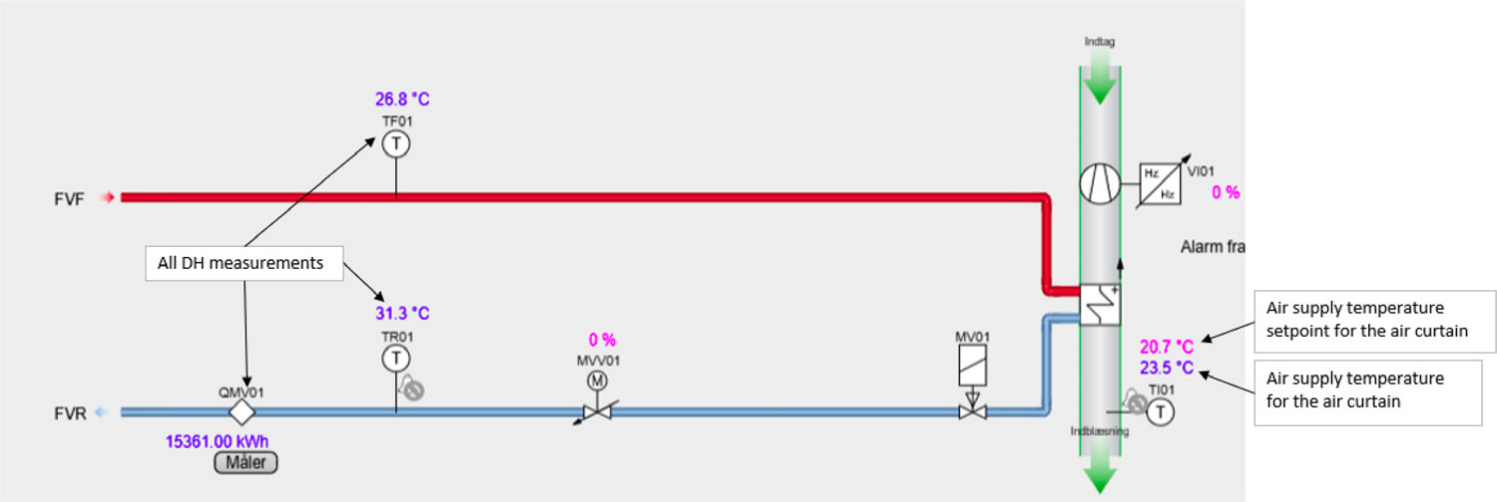
Schematic of the Door01 air curtain system.

The miscellaneous system category only includes the air curtain system seen in Fig. 31. The system uses a direct DH connection without a mixing loop to supply the necessary heating.

#### 4.1.6 Outdoor

The outdoor measurements for temperature and solar radiation are all performed on the building, while the wind speed is measured on a nearby building, meaning that it is the local climate.

#### 4.1.7 Rooms

For the room measurements, the room temperature and CO2 concentration measurements are performed at the control panel, which is generally located next to the door to the room, with the meeting room being different, as the control panels here are located on a wall further away from the door.

**Fig. 32:**
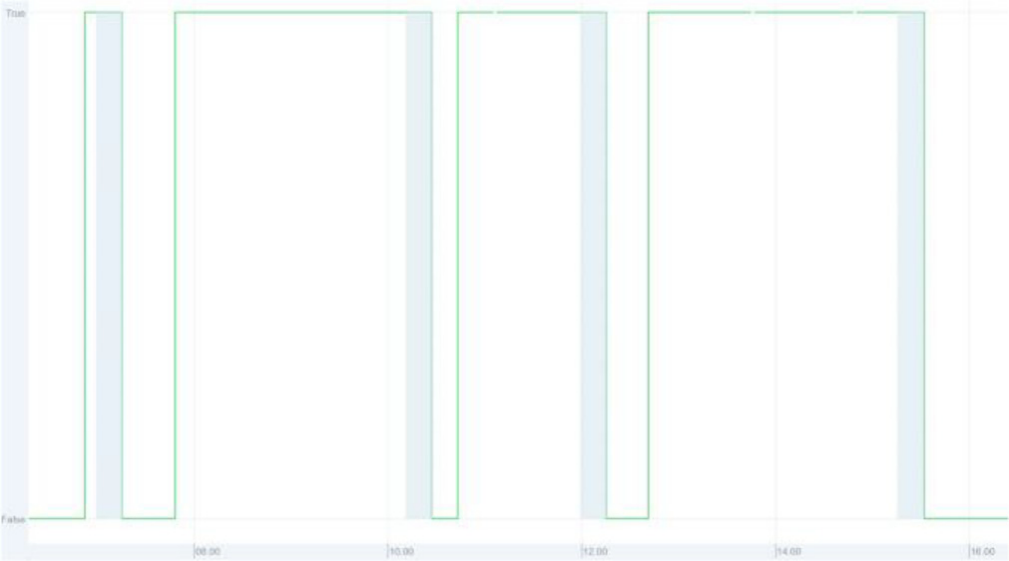
Example of room presence signal. The green line is the signal provided in the dataset. The blue hatched area is the 15-minute delay to remove to get the best estimate for the actual presence.

The presence sensor used in all the rooms is a passive infrared (PIR) type sensor located in the ceiling of the room, normally in the middle if possible. The signal provided is not the direct PIR signal, but instead it is an activity signal, meaning that it has a 15-minute delay from the last time the PIR was active to it showing inactive. The presence signal should therefore have the 15-minute period before the signal becomes false (0) removed to get the best estimate of presence in the room. An example of this is shown in Fig. 32.

All the rooms have Variable Air Volume (VAV) dampers except for the smaller offices towards the north on floor 3, which have Constant Air Volume (CAV) dampers. All the rooms are designed to have balanced ventilation.

### 4.2 NEST pilot

Empa’s NEST demonstrator is a mixed-use vertical energy district. It offers long-term monitoring of rented apartment buildings (3 units as shared flats with 2–3 tenants each, data available back to 2017), office spaces (2 units with 4–8 desks each, 4 meeting rooms in total), and a gym building, to assess real-life use patterns of people, district energy systems, and energy consumption. The demonstrator is equipped with >10,000 high-resolution sensors and energy meters. It offers a versatile neighbourhood-scale multi-energy platform to optimize energy management at the district level. Because of temporary and evolving structure, the NEST building is not certified with a single Minergie label such as Minergie-P-ECO (a Swiss sustainable building standard combining high energy efficiency (Minergie-P) with stringent ecological and healthy construction materials (ECO)) or GEAK (cantonal building energy certificate). Instead, the building envelope follows Minergie-level requirements, and sustainability concepts similar to Minergie-P-ECO are considered in design and operation.

Based on the Horizon Europe HEATWISE project scope for tertiary buildings, we have selected four buildings (UMAR, Sprint, DFAB, and HiLo), which cover both residential and office spaces, for analysis. The data set is structured around these different buildings. Additional building data are available, and further data access and collaboration are possible after registering and requesting access to the NEST database (see https://wiki.nestcloud.ch) [5].

#### 4.2.1 System architecture and data flow

**Fig. 33:**
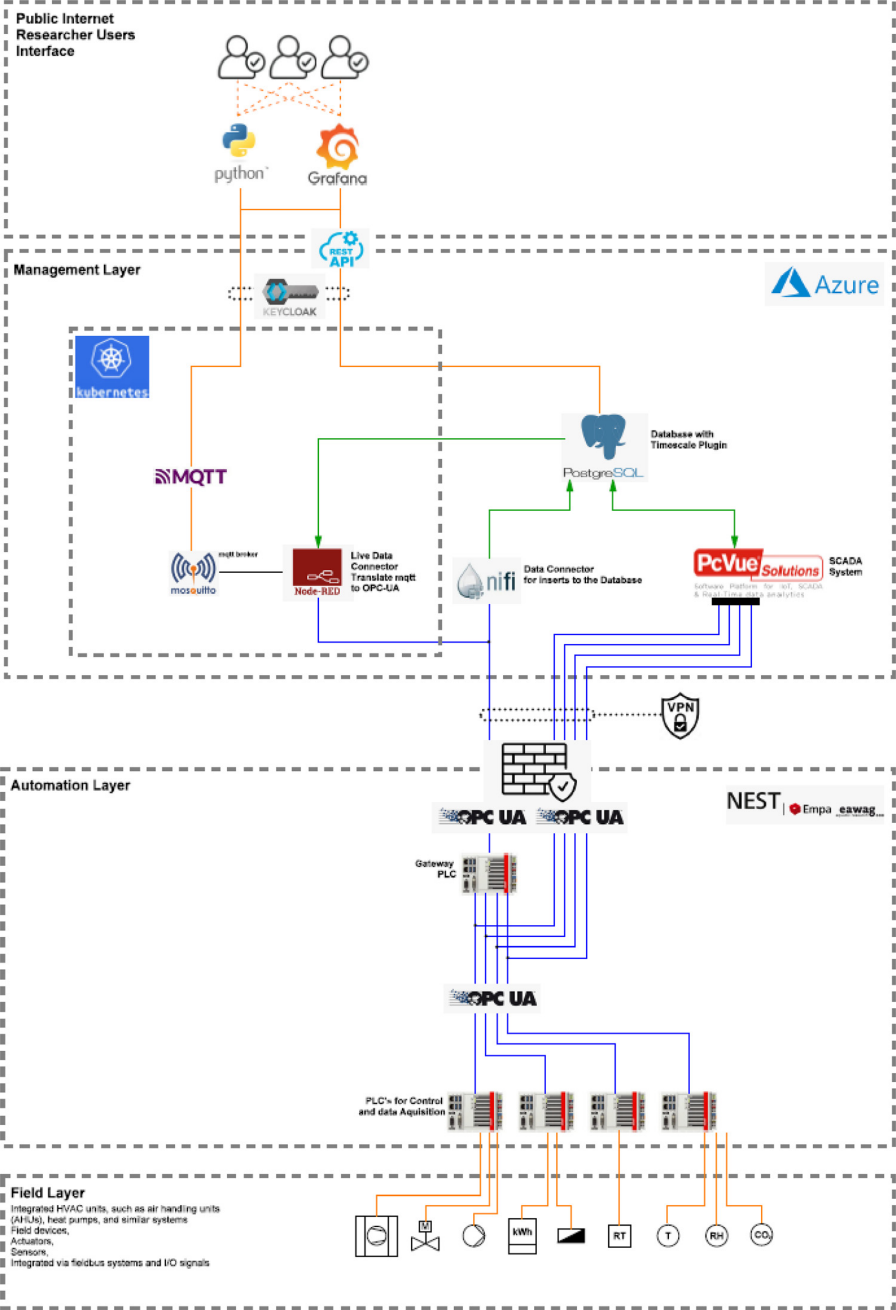
NEST system architecture and data flow.

As illustrated in Fig. 33, the NEST data management system consists of three layers: field, automation, and management. It collects and processes data in a structured way, stores it centrally, and provides secure access for visualization, analysis, and supervisory control. At the field level, sensors, actuators, and embedded devices measure variables such as temperature, humidity, CO₂, and energy consumption. Programmable logic controllers (PLCs) acquire and preprocess these signals. On the automation level, PLCs expose their data via OPC UA servers. A central OPC UA gateway aggregates information from multiple controllers and provides a standardized, vendor-independent data model for interoperable machine-to-IT communication.

Data is transferred to the management level through a secure network connection (firewall/VPN). A NiFi connector ingests the data and stores it in a PostgreSQL database with Timescale extensions, enabling efficient long-term storage and fast queries on historical time-series data. MQTT is used in parallel for lightweight event-driven communication. A Mosquito broker distributes messages, and a Node-RED connector translates between MQTT and OPC UA when needed. Data access is possible either directly through the database for analytical tasks or via a SCADA system (PcVue) for visualization, ensuring an end-to-end data chain from sensors to the management layer.

Researchers access the data through a dedicated user and access layer at the management level. Communication over the public internet is secured, and Keycloak provides centralized authentication, authorization, and role management for both REST APIs and visualization services. A REST API allows authorized users to retrieve time-series and system data programmatically, for example, via Python for scientific analysis or modeling. Grafana offers a web-based interface for dashboards, monitoring, and exploratory data analysis. Both access paths rely on the same time-series database and security mechanisms.

Backend services run in containerized environments (Kubernetes), enabling scalable, reproducible deployments. The architecture supports controlled, role-based remote access to research data while preventing direct access to the automation or field layers, ensuring both IT security and system integrity.

#### 4.2.2 UMAR

**Fig. 34:**
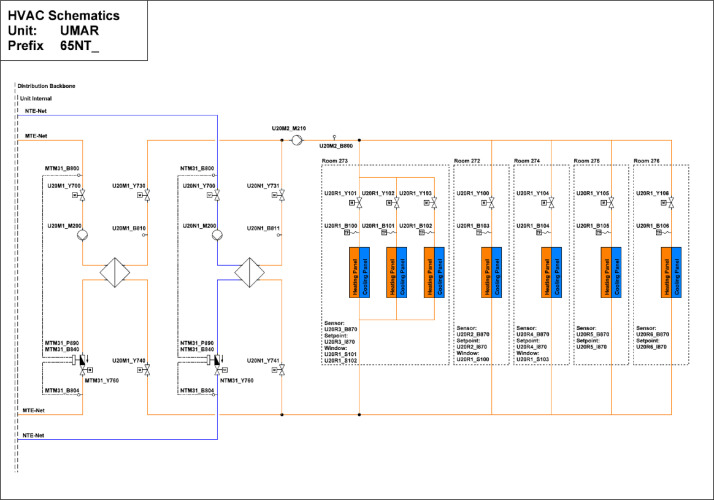
Thermal distribution at UMAR.

The layout of the thermal network is illustrated in Fig. 34. The Unit has large windows, resulting in high exposure to solar irradiance. The heating system primarily supplies thermal energy during winter, drawing heat from the MTE grid via a heat exchanger. Heat is emitted and distributed to the rooms through a ceiling heating system.

**Fig. 35:**
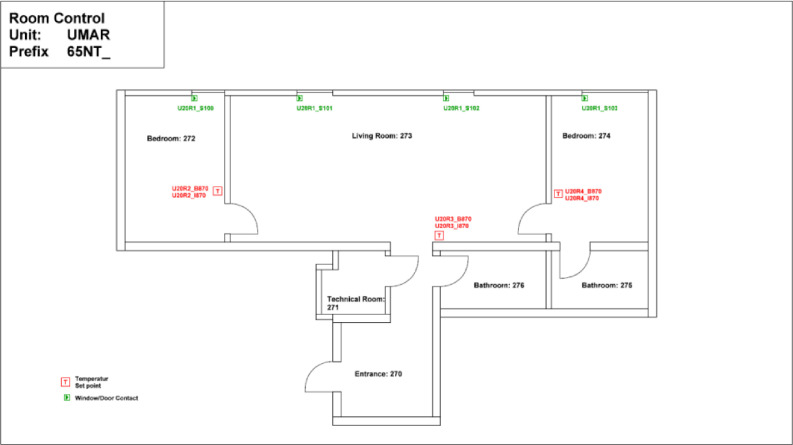
Room control UMAR building.

The location of room control sensors in the UMAR building is illustrated in Fig. 35. It includes sensors for window contact, room temperature, relative humidity, CO2 concentration, brightness, and temperature set-point.

#### 4.2.3 Sprint

**Fig. 36:**
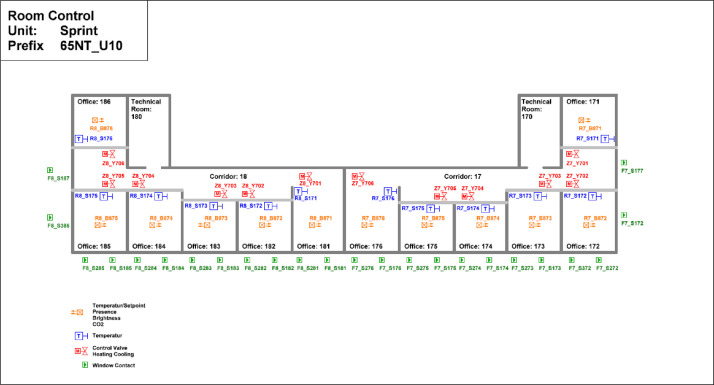
Sensor placement at the Sprint building across the different offices, showing that each office has separate sensors.

**Fig. 37:**
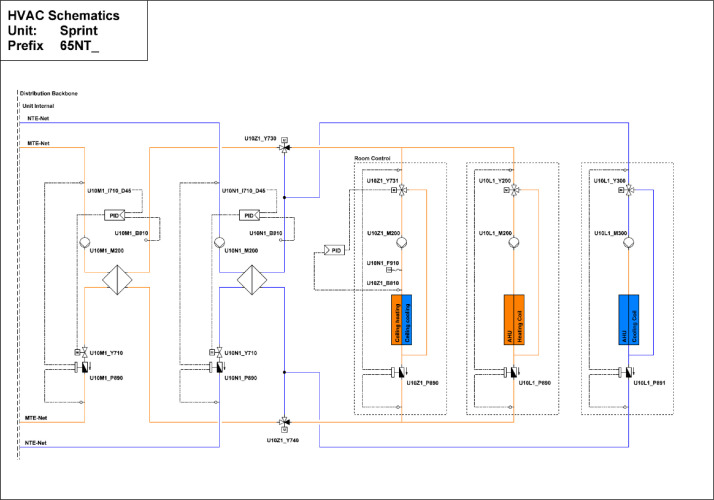
The thermal distribution of the Sprint Unit shows the network for heating and cooling, as well as a separate system for air conditioning and the ceiling heating/cooling system.

The heating system extracts heat from the MTE grid through a heat exchanger. Each office space is equipped with sensors for occupancy and CO_2_ levels. Additionally, temperature setpoints and measured temperature data are available. The layout of the sensors can be seen in Fig. 36, while the thermal distribution is presented in Fig. 37, which shows that the partial air conditioning system is also connected to the thermal network, and in contrast to the ceiling heating/cooling system, there is a separate metering for the partial air conditioning heater and cooler.

#### 4.2.4 DFAB

**Fig. 38:**
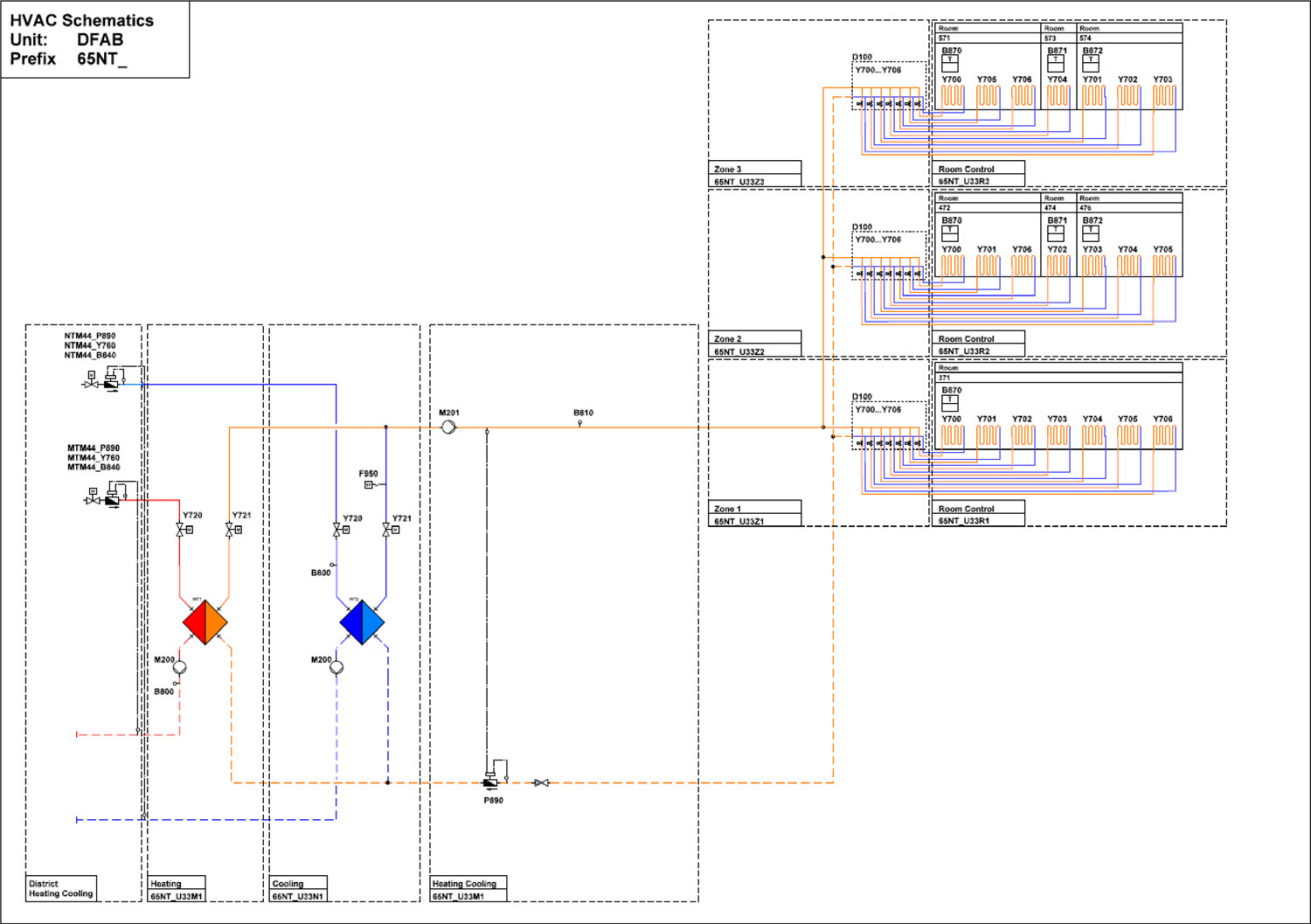
Measurement concept for heating system data in the DFAB building.

Fig. 38 illustrates the various rooms and their corresponding thermal distributions among the three floors of the DFAB building.

**Fig. 39:**
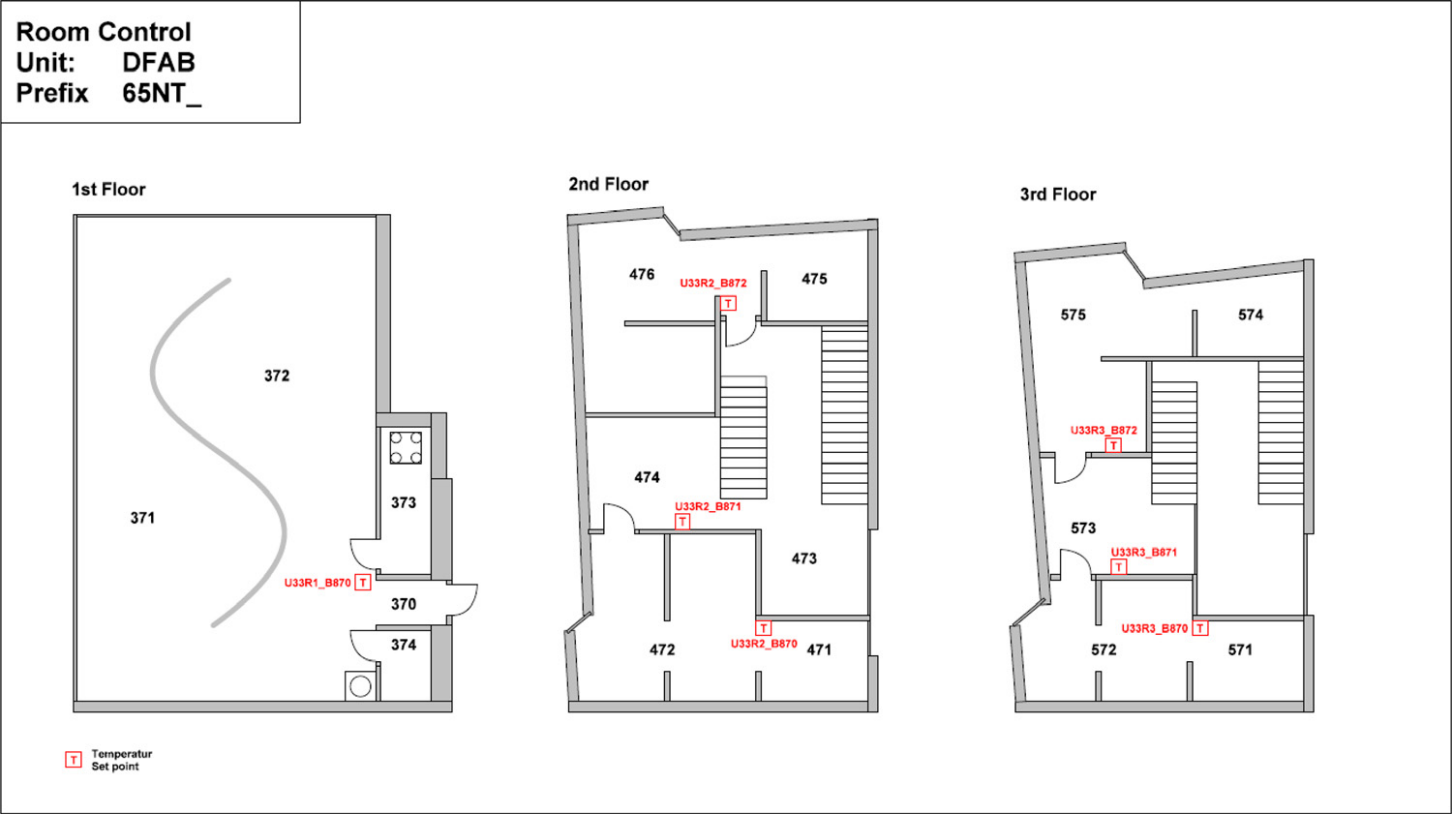
Different rooms with zones on the different floors of the DFAB Unit.

In Fig. 39, the locations of the room sensors and the corresponding room names are visible. For the DFAB Unit, there are no presence or CO2 sensors available.

#### 4.2.5 HiLo

**Fig. 40:**
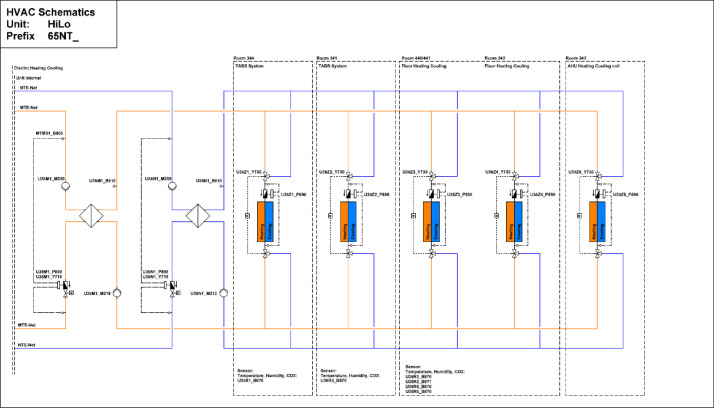
Measurement concept for heating system data for HiLo building.

**Fig. 41:**
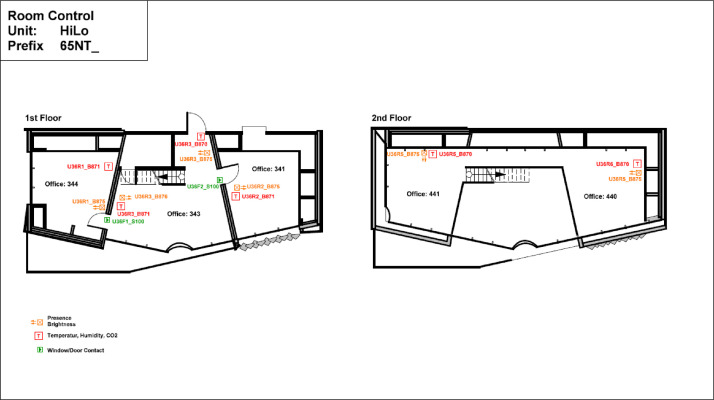
Room control HiLo building.

The heat is sourced from the MTE grid. The heating system is hydraulically decoupled from the MTE grid with a heat exchanger, as seen in Fig. 40. The heat is distributed through the ceiling system and underfloor systems. Offices 1 and 2 feature ceiling heating with TABS, while the gallery and main space utilize floor heating. For HiLo, sensor data are available for CO_2_ levels, occupancy status, brightness, and temperature. The layout of sensor placements is shown in Fig. 41. Additionally, rooms 341 and 344 are equipped with door-opening sensors, as shown in the same figure.

## Limitations

### AAU pilot

Due to difficulties with the building management system in the early part of 2023, some data points were missing until it could be restarted properly. An overview of this is shown in the data availability images.

### NEST pilot

The data set covers the period from November 2023 to July 2024. During this time, not all sensors had been installed. For the Sprint unit, PV generation data is not included. For UMAR, no CO₂ data is available for this period. Some missing values appear as the last observed value. In addition, domestic hot water data in the high-temperature network is incomplete due to a malfunctioning sensor.

## Ethics Statement

The authors have read and followed the ethical requirements for publication in Data in Brief and confirm that the current work does not involve human subjects, animal experiments, or any data collected from social media platforms.

## CRediT Author Statement

**Simon Pommerencke Melgaard:** Conceptualization, Methodology, Software, Validation, Formal Analysis, Investigation, Data Curation, Writing – Original Draft, Writing - Review & Editing, Visualization, Project Administration. **Thomas Juul:** Investigation, Data Curation, Writing – Review & Editing. **Rasmus Lund Jensen:** Conceptualization, Resources, Writing – Review & Editing, Supervision, Project Administration, Funding Acquisition. **Alessandro Tell:** Conceptualization, Methodology, Software, Validation, Formal Analysis, Investigation, Data Curation, Writing – Original Draft, Writing - Review & Editing, Visualization. **Gabriele Humbert:** Conceptualization, Resources, Writing – Review & Editing, Supervision, Funding Acquisition. **Reto Fricker:** Data Acquisition, Software, Validation. **Sascha Stoller**: Data acquisition, Software, Validation. **Marco Kreyenbuehl:** Data Curation, Visualization. **Philipp Heer:** Writing – Review & Editing, Project Administration, Funding Acquisition. **Binod Koirala:** Conceptualization, Resources, Writing – Review & Editing, Supervision, Project Administration, Funding Acquisition.

## Data Availability

ZenodoA high-resolution dataset for a Danish educational building and Swiss multipurpose building with submeter electricity and heating measurements, room measurements, and outdoor measurements (Original data).
